# Facile Synthesis of Catechol-Containing Polyacrylamide Copolymers: Synergistic Effects of Amine, Amide and Catechol Residues in Mussel-Inspired Adhesives

**DOI:** 10.3390/polym15183663

**Published:** 2023-09-06

**Authors:** Lorand Bonda, Janita Müller, Lukas Fischer, Maryna Löwe, Alexej Kedrov, Stephan Schmidt, Laura Hartmann

**Affiliations:** 1 Institut für Organische und Makromolekulare Chemie, Heinrich-Heine-Universität Düsseldorf, Universitätsstr. 1, 40225 Düsseldorf, Germany; bolor100@hhu.de (L.B.); janita.mueller@hhu.de (J.M.); 2 Lehrstuhl für Technische Chemie II, Universität Duisburg-Essen, Universitätsstr. 7, 45141 Essen, Germany; lukas.fischer@uni-duesseldorf.de; 3 Synthetische Membransysteme, Institut für Biochemie, Heinrich-Heine-Universität Düsseldorf, Universitätsstr. 1, 40225 Düsseldorf, Germany; loewe@hhu.de (M.L.); kedrov@hhu.de (A.K.); 4 Institut für Makromolekulare Chemie, Albert-Ludwigs-Universität Freiburg, Stefan-Meier-Str. 31, 79104 Freiburg, Germany

**Keywords:** mussel foot proteins (Mfps), free radical polymerization, underwater adhesive, DOPA, QCM, ellipsometry

## Abstract

The straightforward synthesis of polyamide-derived statistical copolymers with catechol, amine, amide and hydroxy residues via free radical polymerization is presented. In particular, catechol, amine and amide residues are present in natural mussel foot proteins, enabling strong underwater adhesion due to synergistic effects where cationic residues displace hydration and ion layers, followed by strong short-rang hydrogen bonding between the catechol or primary amides and SiO_2_ surfaces. The present study is aimed at investigating whether such synergistic effects also exist for statistical copolymer systems that lack the sequence-defined positioning of functional groups in mussel foot proteins. A series of copolymers is established and the adsorption in saline solutions on SiO_2_ is determined by quartz crystal microbalance measurements and ellipsometry. These studies confirm a synergy between cationic amine groups with catechol units and primary amide groups via an increased adsorptivity and increased polymer layer thicknesses. Therefore, the free radical polymerization of catechol, amine and amide monomers as shown here may lead to simplified mussel-inspired adhesives that can be prepared with the readily scalable methods required for large-scale applications.

## 1. Introduction

**Figure 1:**
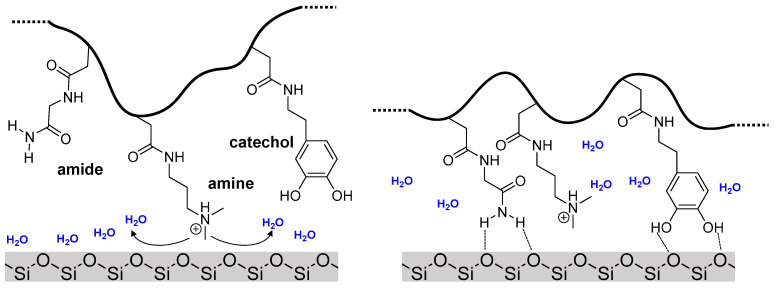
Charged amine residues on polymers as synthesized in this work displace the ion layer and hydration layer on SiO_2_ surfaces (**left**), thereby enabling close range hydrogen bonding of catechol or amide units (**right**).

Underwater adhesion is significantly limited by hydration layers and associated salt ions that prevent the adhesive groups’ direct contact with the surfaces of the materials [1,2]. Marine adhesive proteins secreted by barnacles, sandcastle worms, mussels and similar organisms nevertheless show excellent binding to inorganic and organic surfaces, even in the presence of high salt concentrations [3,4]. Particularly for mussels, sticky proteins known as mussel foot proteins (Mfps) have evolved that get around this issue by displacing the hydration layers and surface salts before bridging to surfaces via strong bonding, primarily through L-3,4-dihydroxyphenylalanine (DOPA) groups [5,6,7]. The catechol units of DOPA bind to minerals using short-range bidentate hydrogen bonding via the hydroxy groups. According to recent findings, the presence of DOPA close to the cationic amino acids lysine and arginine is crucial for strong binding. Indeed, mussel adhesion proteins comprise a large amount of DOPA and amine residues. For example, Mfp-5 contains 30 mol% DOPA and 28 mol% cationic residues that are usually in close proximity along the protein chain [8]. The cationic residues are capable of displacing the hydration and salt layer and assisting the catechol residues in binding to the surface (Figure 1). The synergy between catechol and charged groups was confirmed using various adhesion assays [8,9,10,11,12,13] and led to the development of various bioinspired adhesive polymers, coatings and hydrogels [3,14,15,16,17,18,19,20,21,22,23,24,25,26,27,28,29,30,31,32,33,34,35,36].

Besides cationic and catechol residues, primary amides in the form of asparagine are another type of residue often present at higher than 10 mol% (in Mfp-2, Mfp-3, Mfp-4 and Mfp-6) [37,38,39,40]. The role of asparagine in Mfps is not entirely understood, but it could be argued that its “helix-breaker” function ensures disordered coil-like conformations to increase the accessibility of the adhesive groups. Importantly, however, for Mfp-3, the amide residues are mostly positioned next to amine and DOPA residues, pointing toward a more sophisticated role of the primary amides [40]. Mfp-3 most likely serves as the adhesion primer, i.e., Mfp-3 binds to the mineral surface and then links to the other Mfps [6,41]. Therefore, we have recently studied the adhesive properties of short sequence-defined oligomers containing catechol, amide and amine residues at different positions [42]. These studies confirmed a significant adhesion enhancement in the case of adjacent catechol and amine groups, but also amide groups were able to strongly increase the adhesion when positioned next to catechol units. Additional hydrogen bonding, favorable conformations or the partially ionic character may explain the observed amide–catechol synergy, but the precise mechanism still awaits detailed analysis.

Nevertheless, to first test the potential benefit of the primary amide function for underwater adhesion, here we establish the synthesis of polyacrylamide-derived statistical copolymers with catechol, amine, amide and hydroxy side chain residues and investigate their adsorption to SiO_2_ surfaces. We focus on free radical polymerization, which is often preferred for larger scale synthesis and applications. Various studies showed the feasibility of free and controlled radical polymerization routes toward uncharged [43,44,45,46,47] and charged [24,30,34,35] catechol-containing copolymers. Importantly, however, the effect of additional primary amide units was not yet studied in such copolymer systems.

## 2. Materials and Methods

Acetone and ethanol were purchased from Carl Roth. Acetonitrile, 2,2-dimethoxypropane, *p*-toluenesulfonic acid, tetrahydrofuran and sodium chloride (98%) were purchased from Sigma Aldrich (Taufkirchen, Germany). Acryloyl chloride (96%) was purchased from Merck (Darmstadt, Germany). Azobis(isobutyronitril) (98%), triethylamine and trifluoroacetic acid were purchased from Acros Organics (Geel, Belgium). Dichlormethane, diethylether, ethyl acetate, methanol, hexane and dimethylformamide were purchased from VWR Prolabo (Darmstadt, Germany). Dopamine hydrochloride (99.96%) and glycinamide hydrochloride were purchased from BLD Pharmatech (Kaiserslautern, Germany). Hydroquinone was purchased from J.T. Baker (Phillipsburg, NJ, USA). Potassium carbonate, lithium hydroxide and magnesium sulfate were purchased from Fisher Scientific. Methyl trifluoroacetate was purchased from Fluorochem (Hadfield, UK). *N*-[3-(Dimethylamino)propyl]acrylamide (98%) and *N*-(2-hydroxyethyl)acrylamide (98%) were purchased from TCI (Tokyo, Japan). Sodium hydrogencarbonate, hydrochloric acid (37%) and toluene were purchased from VWR Chemicals.

### 2.1. ^1^H NMR

^1^H NMR spectra were recorded at room temperature with a Bruker AVANCE III 300 (Hercules, CA, USA) (for 300 MHz) and 600 (for 600 MHz). The chemical shifts were reported relative to solvent peaks (chloroform and water) as internal standards and reported as δ in parts per million (ppm). Multiplicities were abbreviated as s for singlet, d for doublet, t for triplet and m for multiplet.

### 2.2. Size Exclusion Chromatography-Multi-Angle Light Scattering (H_2_O-SEC-MALS)

SEC analysis was conducted with an Agilent 1200 series HPLC system and three aqueous SEC columns provided by Polymer Standards Service (PSS). The columns were two Suprema Lux analytical columns (8 mm diameter and 5 μm particle size) and one precolumn (50 mm, 2 × 160 Å of 300 mm and 1000 Å of 300 mm). The eluent was a buffer system consisting of MilliQ water and 30% acetonitrile with 50 mM, NaH_2_PO_4_, 150 mM NaCl and 250 ppm NaN_3_ with a pH = 7.0 (via addition of 50 mL 3 molar aqueous sodium hydroxide solution) filtered with inline 0.1 μm membrane filter and running at 0.8 mL per minute. Multi-angle light scattering was recorded via miniDAWN TREOS and differential refractive index spectra with Optilab rEX, both supplied by Wyatt Technologies EU (Dernbach, Germany). Data analysis was conducted with Astra 5 software and a dn/dc value of 0.156 for each polymer.

### 2.3. Dimethylacetamide-Size Exclusion Chromatography (DMAc-SEC)

SEC analysis was conducted with an SEC column provided by Polymer Standards Service (PSS). The column was a PSS GRAM linear column (8 mm diameter and 10 μm particle size), and a Jasco PU-2080 pump was used (Easton, MD, USA). The eluent was dimethylacetamide running at 1 mL per minute and the measuring temperature was 60 °C. Differential refractive index spectra were recorded with an ETA-2020 RI detector supplied by WGE BURES GmbH & Co. KG (Dallgow-Döberitz, Germany).

### 2.4. Freeze-Drying

Lyophilization was performed with an Alpha 1-4 LD instrument from Martin Christ Freeze Dryers GmbH. A temperature of –42 °C and a pressure of 0.1 mbar were maintained throughout the freeze-drying process.

### 2.5. High Pressure Liquid Chromatography (HPLC)

RP-HPLC/MS (Reversed Phase-HPLC/Mass Spectroscopy) was performed on an Agilent Technologies 1260 Infinity System using an AT 1260 G4225A degasser, G1312B binary pump, G1329B automatic liquid sampler, G1316C thermostatted column compartment, G1314F variable wavelength detector at 214 nm and an AT 6120 quadropole containing an electrospray ionisation (ESI) source. The mobile phase consisted of buffer A (water:acetonitrile 95:5 (*v*/*v*), 0.1 vol.% formic acid) and buffer B (water:acetonitrile 5:95 (*v*/*v*), 0.1 vol.% formic acid). HPLC runs were performed on a Poroshell 120 EC-C18 (3.0 × 50 mm, 2.5 μm) RP column from Agilent at a flow rate of 0.4 mL/min 95% buffer A and 5% buffer B (0–5 min), following a linear gradient to 100% buffer B (5–30 min) at 25 °C. ESI-MS for GlcNAc-oligomers and sulfates was performed using 95% buffer A and 5% buffer B without formic acid and a fragmentor voltage of 40–60 V (m/z range of 200 to 2000).

### 2.6. Elllipsometry

Ellipsometry was conducted with a Sentech SI-SE 800 spectroscopic ellipsometer (Sentech Instruments GmbH, Berlin, Germany) on silicon wafers (Science Services, Munich, Germany). For surface preparation, the wafers were treated in a 5:1:1 mixture of ultrapure water, hydrogen peroxide (30%) and ammonia (25%) at 70 °C for 30 min, followed by rinsing with ultrapure water. Next, the wafers were immersed in the polymer solutions containing 0.1 M NaCl for 20 min followed by immersion in 0.1 M NaCl solutions to remove unbound polymer, rinsing with pure water and drying. For ellipsometry data evaluation, the thickness of the naturally grown oxide layer was determined on uncoated wafers from the same batch (12 ± 0.3 nm) and the refractive index of the polymer layer was assumed to be 1.5 [48]. This allowed for the determination of the polymer layer thickness as the only free parameter.

### 2.7. Quartz Crystal Microbalance Measurements

QCM measurements were performed with a QCM-D instrument qCell T Q2 (3T analytic GmbH, Neuhausen ob Eck, Germany) with dual sensor channels equipped with quartz chips from the same company. The chips were activated by air plasma treatment for 30 s before use. A solution of 0.1 M NaCl was prepared, filtered (pore size 0.1 µm) and degassed for 20 min. The polymers were dissolved in the solution at a concentration of 0.1 mg/mL. Before the samples were injected, the chips were stabilized using a 0.1 M NaCl solution without polymer. Polymer samples were injected with a flow rate of 80 µL min^−1^ for 1000 s followed by the pumping of the pure NaCl solution for 1500 s. QCM chips were regenerated after each run by first placing them in an SDS bath for 30 min and then treating them with piranha solution (H_2_SO_4_:H_2_O_2_ 30%, 3:1, *v*/*v*) for 2 min.

## 3. Results and Discussion

### 3.1. Synthesis of Monomers

**Scheme 1:**
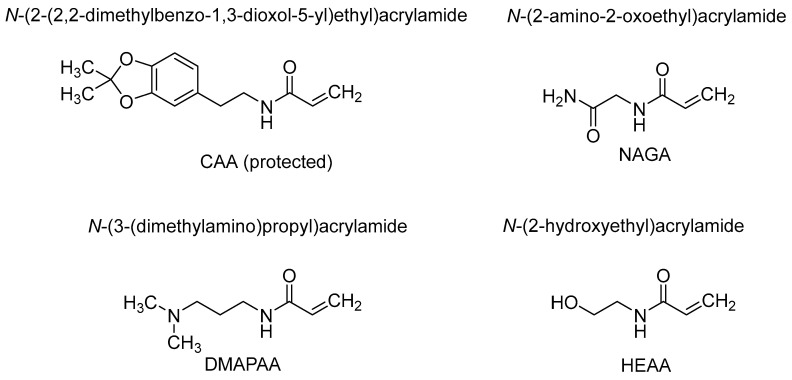
The different *N*-substituted acrylamide monomers used to build the polymers.

The polymers to be synthetized were supposed to be varied in their overall composition of catechol, amine and amide groups, as these are thought to be the main contributors to the underwater adhesion of Mfps. Here we aimed at acrylamide-derived polymers owing to their well-established solution polymerization procedures and frequent use in functional materials. Therefore, the following four *N*-substituted acrylamide monomers were used: catechol-containing acrylamide (CAA), amide-containing acrylamide (PA), tertiary amine-containing acrylamide (TA) and hydroxy-containing acrylamide (HY) (Scheme 1).

The CAA and PA monomers were prepared synthetically (see Supplementary Materials Chapter S1), while the TA and HY monomers were purchased commercially. Special focus was devoted to the synthesis of the protected catechol-bearing monomer CAA since catechols are prone to side reactions, which may lead to undesired crosslinking of the polymers. Here we use the acetonide-protection of the catechol monomer and make it suitable for radical polymerization and the release of the free catechol by deprotection after successful polymerization.

**Scheme 2:**
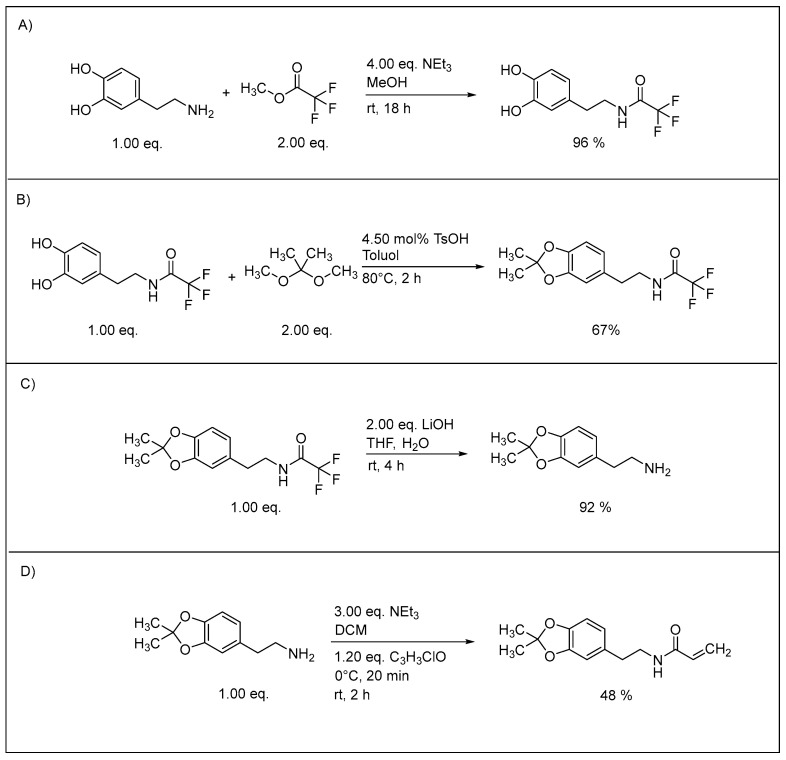
Synthesis of the catechol monomer CAA. Attaching the methyltrifluoracetate protecting group to the amine (**A**), attaching the acetonide protecting group (**B**), deprotecting the methyltrifluoracetate goup (**C**), syntheses of the final CAA monomer (**D**).

The synthesis of CAA was carried out in four steps (Scheme 2). The first three steps were developed according to synthesis known from the literature [49,50]. Dopamine hydrochloride was used as the starting material. Before the acetonide protecting group was attached to the catechol, the primary amine of dopamine first had to be protected with a trifluoroacetyl group using methyl trifluoroacetate. If the acetonide protecting group was introduced directly, an undesirable Pictet–Spengler condensation would otherwise occur and a tetrahydroisoquinoline species would be obtained. This reaction occurs frequently with phenylamines, such as dopamine, in the presence of aldehydes or ketones [51]. In the second step, the acetonide protecting group was attached using 2,2 dimethoxypropane, the ketal of acetone. This is an important step because catechols can be easily oxidized to quinones by atmospheric oxygen even at neutral to weakly alkaline pH [52]. Similarly, without an acetonide protecting group, undesirable crosslinking of the dopamine acrylamide monomers could occur during free radical polymerization. The introduced protecting groups are orthogonal to each other, meaning they can be cleaved off independently: The acetonide protecting group is removed under acidic conditions and the trifluoroacetyl protecting group is removed under basic conditions. After removing the trifluoroacetyl group, the resulting dopamineacetonide was reacted with acryloyl chloride to give the final monomer.

**Scheme 3:**
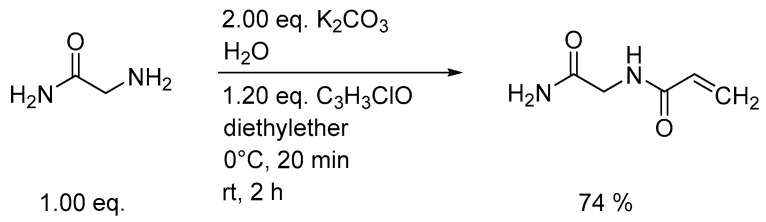
Reaction conditions for the synthesis of the N-acryloylglycinamide (PA) monomer.

The synthesis of the primary amide-bearing PA monomer (Scheme 3) was adapted from Lutz et al. with minor changes in the purification protocol (see Supplementary Information Chapter S1) [53]. The choice of using PA for the introduction of primary amide side chain residues was inspired by asparagine, as is found in some Mfps.

### 3.2. Polymer Synthesis

The objective was to study the polymer adsorption on silica or glass surfaces at different monomer compositions. Therefore, the monomers were copolymerized at different ratios, followed by deprotection for all CAA-containing polymers (designated CA in deprotected form). The optimal reaction conditions were determined by the variation of the polymerization parameters and analyzing the isolated polymers with regard to monomer incorporation, number-average molecular weights and dispersities (see Supplementary Materials, Section S2 for details). We focused on varying the CA and PA units which are suspected to increase adhesion via hydrogen bonding. Additionally, the amount of TA units was varied to enable the synergistic effects of the cations helping to displace the water and salt barrier. All polymers were polymerized with at least 50% of the non-adhesive “filler” monomer HY. The variation of the monomers incorporated into the target polymers and the monomer fractions selected in these allow conclusions to be drawn on the influence of the monomer interactions with each other, as well as on the influence of the monomer fractions incorporated in each case on the polymer adhesion. Overall, eight polymers with different monomer incorporation were synthesized (Table 1). In the sample code, the filler monomer HY is omitted for clarity.

Two copolymers, TA52 and PA50, consisting of two monomers were prepared (Table 1). The main reason for their synthesis was to obtain adhesion values for polymers without catechol units for comparison. Three copolymers were synthesized with three monomers: TA15-PA13, CAA4-TA48 and CAA3-PA45. Here the intention was to be able to test the effect of the added catechol groups and to compare the potential synergy with amide and amine groups. Finally, three copolymers containing all four monomers were synthesized. The copolymers CAA4-TA22-PA17 and CAA5-TA5-PA7 were synthesized to study the effect of different TA and PA content on adhesion. The copolymer CAA13-TA15-PA18 can be used to test the extent to which adhesion changes when the catechol moiety is increased relative to the other two comonomers. Since all monomers were incorporated in all three products, this allows interactions between all three residues and the resulting properties of the polymers to be studied. All isolated polymers were obtained as colorless or yellowish solids after purification by dialysis (MWCO 7.50 kDa) followed by lyophilization.

### 3.3. Acetonide Deprotection

In order to obtain catechol units for adhesion studies, the acetonide protecting group was removed from the CAA-containing copolymers right before performing the adhesion studies, so oxidation of the catechol hydroxyl groups does not occur. Successful and complete removal of the protecting groups was confirmed by ^1^H NMR (see Supplementary Information, Figures S23–S27).

### 3.4. Adsorption to Quartz Surfaces

**Figure 2:**
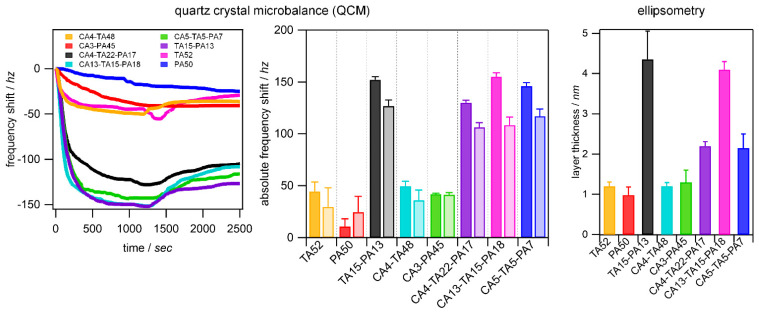
Polymer adsorption measured by QCM and ellipsometry on quartz surfaces. (**Left**): QCM-frequency traces for polymer samples. The dashed line at 1000 sec signifies the region of injecting polymer solutions (0–1000 s) and pumping pure buffer (1000–2500 s). (**Center**): Frequency shifts from 0 s to 1000 s (fully colored bars) and frequency shifts at 2500 s (equilibrium, light colors) after rinsing with pure NaCl solution. (**Right**): Polymer film thicknesses on quartz chips after adsorption, rinsing and drying measured by ellipsometry.

To determine the interaction of the polymers with SiO_2_ surfaces, quartz crystal microbalance (QCM) and ellipsometry were used. Polymer solutions were prepared in 10 mM phosphate buffer (pH 7.0) containing 0.1 M NaCl. For QCM measurements, polymer solutions were injected at a constant rate for 1000 s followed by pumping pure, polymer-free solutions for another 1500 s to study the equilibrium polymer adsorption. The QCM-frequency traces are shown in Figure 2. To rule out variations by different chips, the measurements were performed using a single QCM chip that was regenerated by piranha solution after each run. Selected samples were analyzed by fresh chips showing similar frequency shifts. Via rinsing with pure NaCl solution after polymer adsorption, the frequency shifts decrease by roughly 5–10% for all polymer samples showing the fraction of loosely bound polymers. For determining the thickness of the polymer films as an alternative measure of polymer adsorption, ellipsometry was used. Silicon chips with naturally grown SiO_2_ layers were immersed in the same polymer solutions for 20 min, followed by rinsing with pure buffer and ultra-pure water. For selected samples, the adsorption time was increased to 40 min, giving similar results compared to 20 min adsorption, confirming that the polymer layer formation was finished after 20 min.

Overall, ellipsometry and QCM measurements showed similar trends: only when combing amide (PA) or catechol units (CA) with the cationic amine groups (TA) was the adsorption strong. When compared to previous results, [42] again confirms that the amine groups can synergize with catechol groups or primary amide groups to strongly bind to glass surfaces. The apparent exception was CA4-TA48 (amine and catechol), which showed low adsorption similar to TA52 (only amines), whereas TA15-PA13 (amide and amine) showed much higher adsorption. This is because TA52 and CA4-TA48 have a very high charge density due to the abundance of cationic TA units; thus, the polymers attain a stretched conformation in bulk and in the adsorbed state, leading to comparatively low film thicknesses. Therefore, highly charged polycations appear to adsorb predominantly via ionic interactions and additional hydrogen bonding via catechol had no additional effect on layer thickness. In addition, the molecular weight of CA4-TA48 was roughly three times lower compared to TA15-PA13, which may also add to the reduced layer thickness. Among the polymers that contain all four monomers, the one with the highest combined catechol and amide content (CA13-TA15-PA18) exhibits the highest film thickness and frequency shifts. Nevertheless, without catechols, but with amides and amines (TA15-PA13), adsorption was also strong. This indicates a potential synergy also between amide and amine units for adhesion to SiO_2_ surfaces, which has been largely overlooked in the development of underwater adhesive polymer systems so far. Such synergy in binding to SiO_2_ surfaces agrees well with earlier studies [11,12] on catechol-based mussel-inspired polymers as well as newer results where amide groups were also included. The interactions of amides and glass surfaces could be due to hydrogen bonding, where the primary amides can donate two hydrogens, similar to catechol residues. Furthermore, the zwitterionic resonance structure of the primary amide (25–30% ionic character) [54] may help to displace the salt or hydration layers on the SiO_2_ surface to increase binding. Overall, we cannot conclude on the molecular details of amide vs. catechol-based adhesion, but also the natural mussel foot protein that primes the rock surface for adhesion contains many asparagine units that present primary amides close to catechol or amine units. Thus, the polymers synthesized here by standard radical polymerization present an improved mimetic of the mussel adhesives due to the added primary amides. With the established synthetic platform enabling the large-scale production of these polymers, future studies can focus on applications as well as direct mechanical adhesion tests.

## 4. Conclusions

In summary, we synthesized polyacrylamide-derived copolymers with catechol, amine, amide and hydroxy side chain residues via free radical polymerization and studied their adsorption on negatively charged SiO_2_ surfaces from saline solutions. The developed monomers show a rather homogenous incorporation rate, suggesting the preparation of statistical copolymers. Furthermore, the obtained yields and the stability of the products suggest that well-behaved polymerization routes were established. Intriguingly, we could confirm a synergy between cationic amine groups with catechol units and the primary amide group, which led to increased adsorption on SiO_2_ surfaces. Thus, the simple statistical polyacrylamide prepared mimics crucial features of natural mussel adhesion proteins even without controlling the sequence of the functional groups in detail. Further studies will explore potential applications of these polymers as adhesives and try to shed light on the molecular mechanisms behind a potential synergy of these functional groups, e.g., by mechanical tests and further varying the content of catechol amide and amine groups in the polymers.

## Figures and Tables

**Table 1:** Specified ratio of theoretical monomer incorporation and target polymers obtained. The numbers in the sample code give the percentage of CAA, TA and PA units; HY is omitted.

Final Polymer	Monomer Reaction Ratio [%]				Monomer Incorporation [%] ^a^				$\bar{M}$ _n_	*Đ*	Yield
(Sample Code)	CAA	TA	PA	HY	CAA	TA	PA	HY	[kDa]		[%]
TA52	0	50	0	50	0	52	0	48	23.5	1.97	91
PA50	0	0	50	50	0	0	50	50	50.33	1.36	39
TA15-PA13	0	15	15	70	0	15	13	72	69.9	1.74	93
CAA4-TA48	5	45	0	50	4	48	0	48	21.9	2.61	98
CAA3-PA45	5	0	45	50	3	0	45	52	23.4	1.92	45
CAA5-TA5-PA7	5	5	5	85	5	5	7	83	63.1	1.87	98
CAA4-TA22-PA17	5	22.5	22.5	50	4	22	17	57	64.4	1.73	94
CAA13-TA15-PA18	15	15	15	55	13	15	18	54	50.9	1.12	91

^a^: Determined by ^1^H NMR-spectroscopy.

## Data Availability

The data presented in this study are available in this article and the supplementary materials.

## Supplementary Materials

The following are available online at https://www.mdpi.com/article/10.3390/polym15183663/s1, Figures S1–S7: NMR data and synthesis detail for monomers, Tables S1–S3: Determination of reaction conditions for free radical polymerization, Figures S8–S22, Table S4: NMR and SEC-MALS of the final polymers, Figure S23–S27: Acetonide deprotection of final polymers. Refs. [49,53] are cited in the supplementary materials.
