# Supplementary material for: Facile Synthesis of Catechol-Containing Polyacrylamide Copolymers: Synergistic Effects of Amine, Amide and Catechol Residues in Mussel-Inspired Adhesives
Source: Polymers (Basel). 2023 Sep 6;15(18):3663. doi: 10.3390/polym15183663 (PMC10535631; doi:10.3390/polym15183663)
Supplement: Supplementary file 1 [file polymers-15-03663-s001.zip › polymers-2526767-Supplementary.pdf]

Supporting Information

Contents

1. Monomer synthesis and characterization 2
   N-(3,4-dihydroxyphenethyl)-2,2,2-trifluoroacetamide 2
   N-(2-(2,2-dimethylbenzo-1,3-dioxol-5-yl)ethyl)-2,2,2-trifluoroacetamide 3
   2-(2,2-dimethylbenzo-1,3-dioxol-5-yl)ethan-1-amine 4
   N-(2-(2,2-dimethylbenzo-1,3-dioxol-5-yl)ethyl)acrylamide (CAA monomer) 5
   N-(2-amino-2-oxoethyl)acrylamide 7
2. Determination of reaction conditions for free radical polymerization 8
3. Synthesis and characterization of final polymers 11
   PA50-stat.-HY50 11
   TA52-stat.-HY48 12
   CAA4-stat.-TA48-stat.-HY48 14
   CAA3-stat.-PA45-stat.-HY52 15
   TA15-stat.-PA13-stat.-HY72 17
   CAA-stat.-TA-stat.-PA-stat.-HY 18
4. Acetonide deprotection of final polymers 23
   CAA4-stat.-TA48-stat.-HY48 23
   CAA3-stat.-PA45-stat.-HY52 23
   CAA4-stat.-TA22-stat.-PA17-stat.-HY57 24
   CAA13-stat.-TA15-stat.-PA18-stat.-HY54 25
   CAA5-stat.-TA5-stat.-PA7-stat.-HY83 25

References 26

1. **Monomer synthesis and characterization**

***N*-(3,4-dihydroxyphenethyl)-2,2,2-trifluoroacetamide**

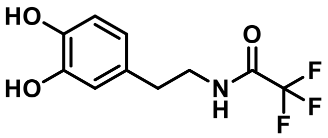

The synthesis procedure of *N*-(3,4-dihydroxyphenethyl)-2,2,2-trifluoroacetamide is known from literature[1] and was carried out according to it. In a round bottom flask, 20 g of dopamine hydrochloride 1 (105.40 mmol) was placed in methanol [2.5 mL/mmol] under an inert gas atmosphere (N_2). After the addition of 21.7 mL of methyl trifluoroacetate (210.80 mmol, 2.00 eq.) and 60 mL of triethylamine (421.60 mmol, 4.00 eq.), the apparatus was purged with nitrogen for an additional five minutes. Methanol was removed at the rotary evaporator and the pH of the crude product was adjusted to 1 by adding one milliliter of concentrated hydrochloric acid. After extraction with ethyl acetate, the organic phase was washed with water and dried with anhydrous magnesium sulfate. Ethyl acetate was removed on rotary evaporator and the product was obtained. A light brownish solid was obtained with a yield of 96%.

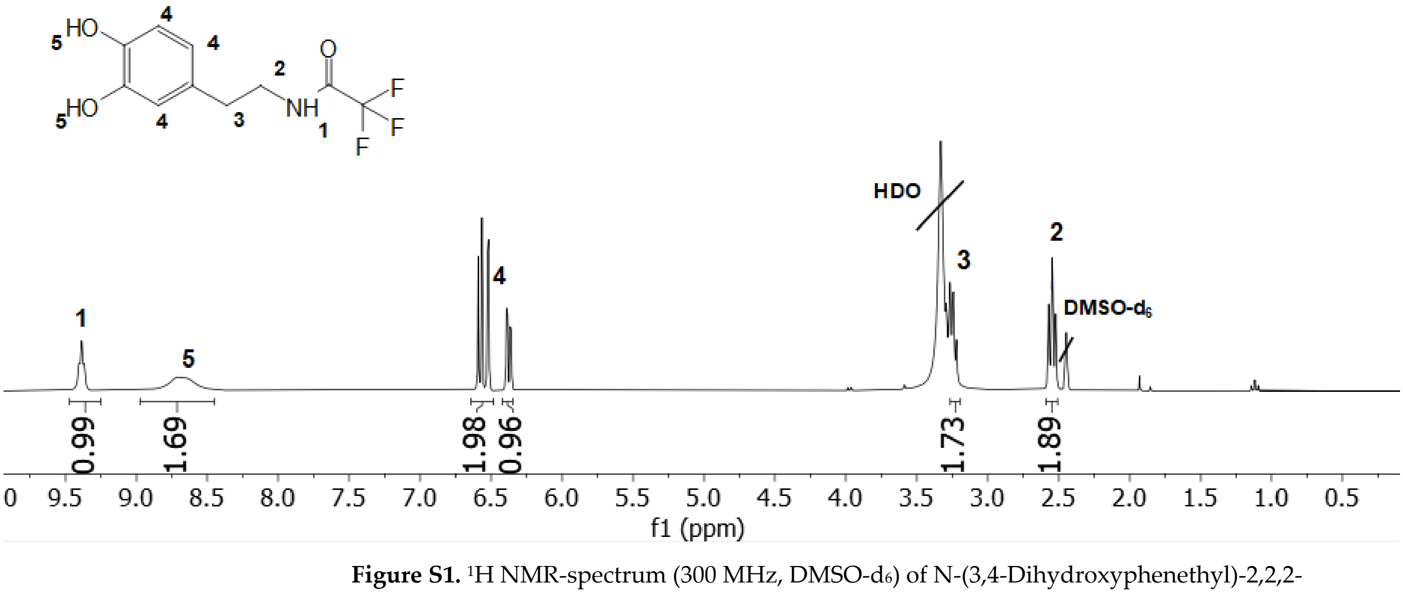

Figure S1. ^1H NMR-spectrum (300 MHz, DMSO-d_6) of N-(3,4-Dihydroxyphenethyl)-2,2,2-trifluoroacetamide.

^1H NMR (300 MHz, DMSO-*d_6*) δ [ppm] 9.39 (t, ^3J = 5.7 Hz, 1H, -N**H**-), 8.68 (s, 2H, -O**H**), 6.65-6.52 (m, 2H, Ar-**H**), 6.37 (dd, ^3J = 8.0, 1H, Ar-**H**), 3.29-3.16 (m, 2H, H_2O overlapping, -C**H**$_2$-NH-), 2.55 (t, ^3J = 7.4 Hz, 2H, -C**H**$_2$-Ar).

N-(2-(2,2-dimethylbenzo-1,3-dioxol-5-yl)ethyl)-2,2,2-trifluoroacetamide

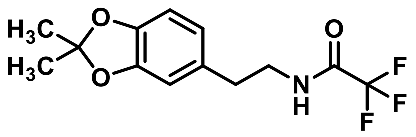

The synthesis procedure of *N*-(2-(2,2-dimethylbenzo-1,3-dioxol-5-yl)ethyl)-2,2,2-trifluoroacetamide is known from literature[1] and was carried out according to it. In a three-neck flask, 25 g of *N*-(3,4-dihydroxyphenethyl)-2,2,2-trifluoroacetamide (100.33 mmol) was placed in toluene [5 mL/mmol] under protective gas atmosphere (N_2). After addition of 24.7 mL of 2,2-dimethoxypropane (200.66 mmol, 2.00 eq.), the reaction solution was degassed for 10 min and heated at 80°C for 15 min under reflux. Then, 775 mg of *p*-toluenesulfonic acid (4.50 mol%) was added and heated at 80°C under reflux for another 2 h. The reaction solution was degassed. The cooled reaction solution was washed three times with water, dried with anhydrous magnesium sulfate, and toluene was removed on the rotary evaporator. The product was obtained as a brownish solid in 67% yield.

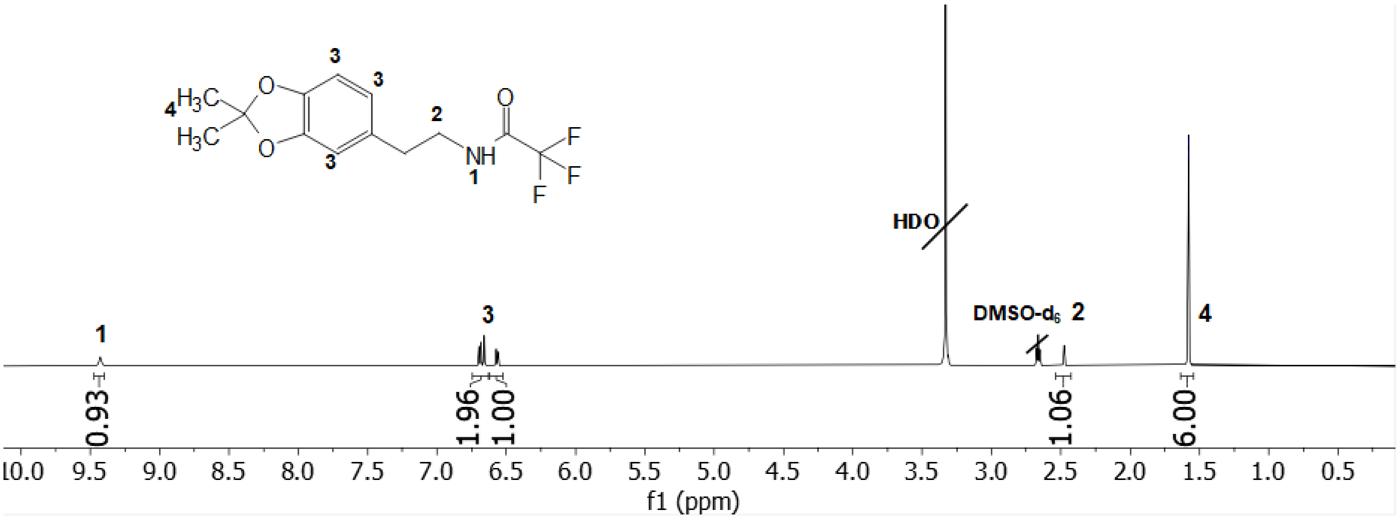

Figure S2. ^{1}H NMR spectrum (600 MHz, DMSO-d$_6$) of *N*-(2-(2,2-dimethylbenzo-1,3-dioxol-5-yl)ethyl)-2,2,2-trifluoroacetamide.

^{1}H NMR (600 MHz, DMSO-*d*$_6$) δ [ppm] 9.43 (t, ^3J = 5.7 Hz, 1H, -N**H**-), 6.72-6.65 (m, 2H, Ar-**H**), 6.57 (d, ^3J = 7.9, 1H, Ar-**H**), 3.30 (m, 2H, -C**H**$_2$-NH- H_2O overlapping), 2.66 (t, ^{3}J = 7.3 Hz, 2H, -C**H**$_2$-Ar-), 1.58 (s, 6H, 2 -C**H**$_3$).

2-(2,2-dimethylbenzo-1,3-dioxol-5-yl)ethan-1-amine

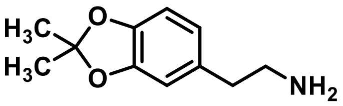

The synthesis procedure of 2-(2,2-dimethylbenzo-1,3-dioxol-5-yl)ethan-1-amine is known from literature[1] and was carried out according to this. 15 g of *N*-(2-(2,2-dimethylbenzo-1,3-dioxol-5-yl)ethyl)-2,2,2-trifluoroacetamide (51.90 mmol) was placed in THF [6 mL/mmol] and 4.32 g of LiOH (103.80 mmol, 2.00 eq.) dissolved in water [2 mL/mmol] was added. The mixture was stirred at RT for 4 h and THF was then removed on the rotary evaporator. The crude product was extracted with ethyl acetate and the organic phase was washed with water. After drying with magnesium sulfate, ethyl acetate was removed with the rotary evaporator. A brownish oil was obtained with a yield of 92%.

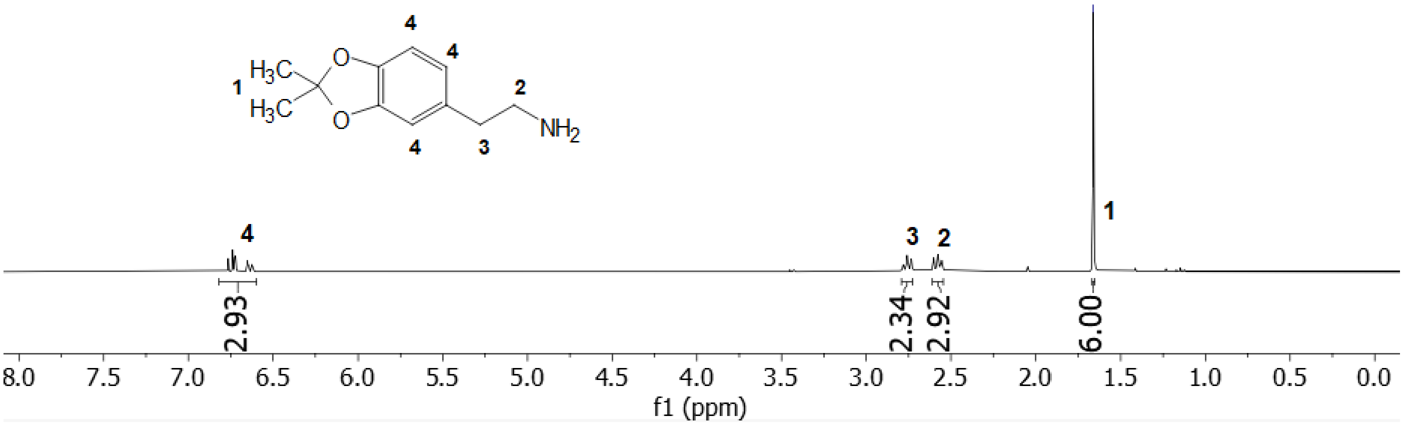

Figure S3. ^1H NMR-spectrum (300 MHz, DMSO-d$_6$) of 2-(2,2-dimethylbenzo-1,3-dioxol-5-yl)ethan-1-amine.

^{1}H NMR (300 MHz, DMSO-*d*$_6$) δ [ppm] 6.84-6.72 (m, 2H, Ar-**H**), 6.64 (d, ^3J = 7.8 Hz), 2.81-2.72 (m, 2H, -C**H**$_2$-NH-), 2.58 (t, 2H, DMSO overlapping, -C**H**$_2$-Ar), 1.66 (s, 6H, 2 -C**H**$_3$).

N-(2-(2,2-dimethylbenzo-1,3-dioxol-5-yl)ethyl)acrylamide (CAA monomer)

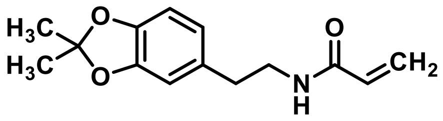

9.8 g dopamine acetonide (50.66 mmol) and 21 mL triethylamine (151.98 mmol, 3.00 eq.) were dissolved in dichloromethane [3 mL/mmol] and cooled to 0°C (ice bath). 4.95 mL of acryloyl chloride (60.79 mmol, 1.20 eq.) was added slowly (about 20 min) to the reaction solution in DCM [1 mL/mmol]. After the addition was complete, the ice bath was removed and the mixture was stirred for 2 h at RT. The reaction solution was washed three times with brine, the organic phase was dried with anhydrous magnesium sulfate, and the dichloromethane was removed on the rotary evaporator. The pressure was set no lower than 600 mBar at 40°C bath temperature so that self-initiated polymerization could not occur. After removal of the solvent, the product was purified by column chromatography. This was done on silica gel using the flash technique, with an eluent mixture consisting of ethyl acetate and *n*-hexane (EE/Hex 1/1 vol.%; Rf = 0.55). The monomer was obtained as a brown highly viscous oil with a yield of 48%.

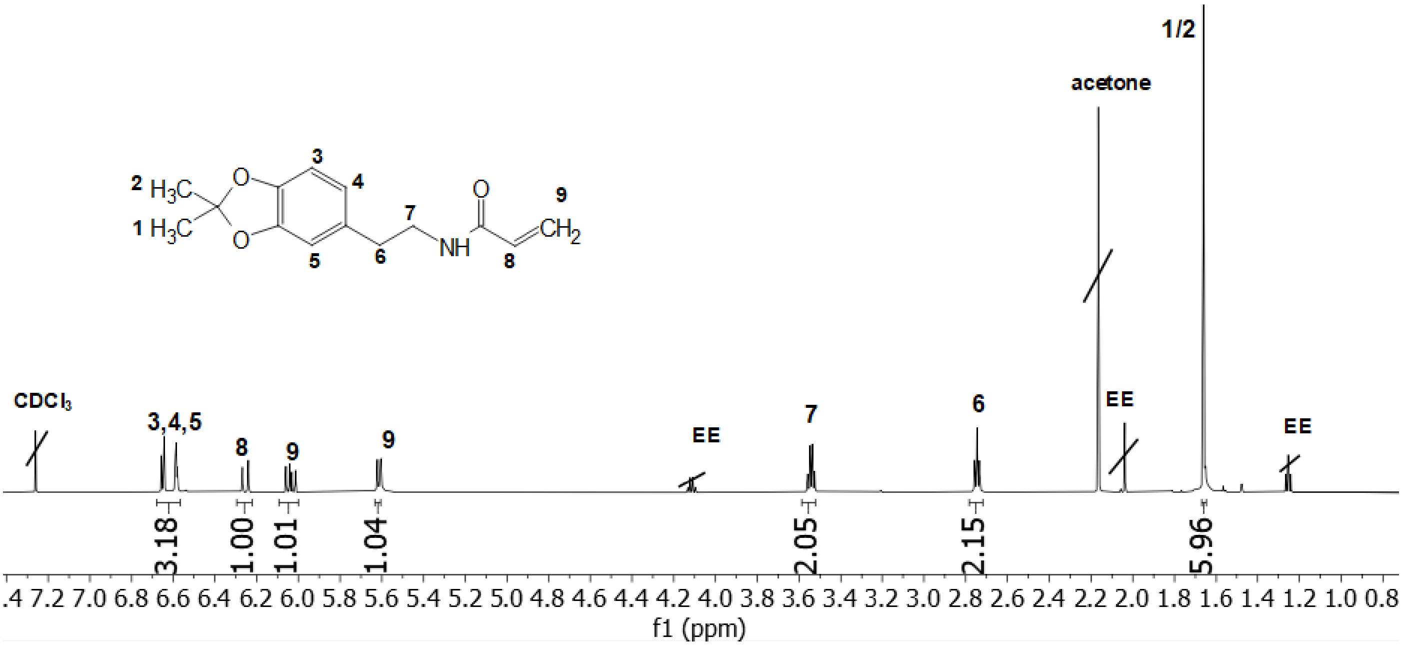

Figure S4. ^{1}H NMR spectrum (600 MHz, CDCl3) of the synthesized dopamineacetonide acrylamide (CAA) monomer.

^{1}H NMR (600 MHz, Chloroform-*d*) δ [ppm] 6.65 (d, J = 8.2 Hz, 2H, Ar-**H**), 6.60-6.58(m, 1H, Ar-**H**), 6.25 (dd, J = 16.9, 1.4 Hz, 1H, -**CH**=CH$_2$), 6.04 (dd, J = 16.9, 10.3 Hz, 1H, =**CH$_2$**), 5.61 (dd, J = 10.3, 1.4 Hz, 1H, =**CH$_2$**), 3.54 (td, ^{3}J = 6.9 Hz, 2H, -**CH$_2$**-NH-), 2.75 (t, ^{3}J = 6.9 Hz, 2H, -**CH$_2$**-Ar), 1.66 (s, 6H, 2 -**CH$_3$**).

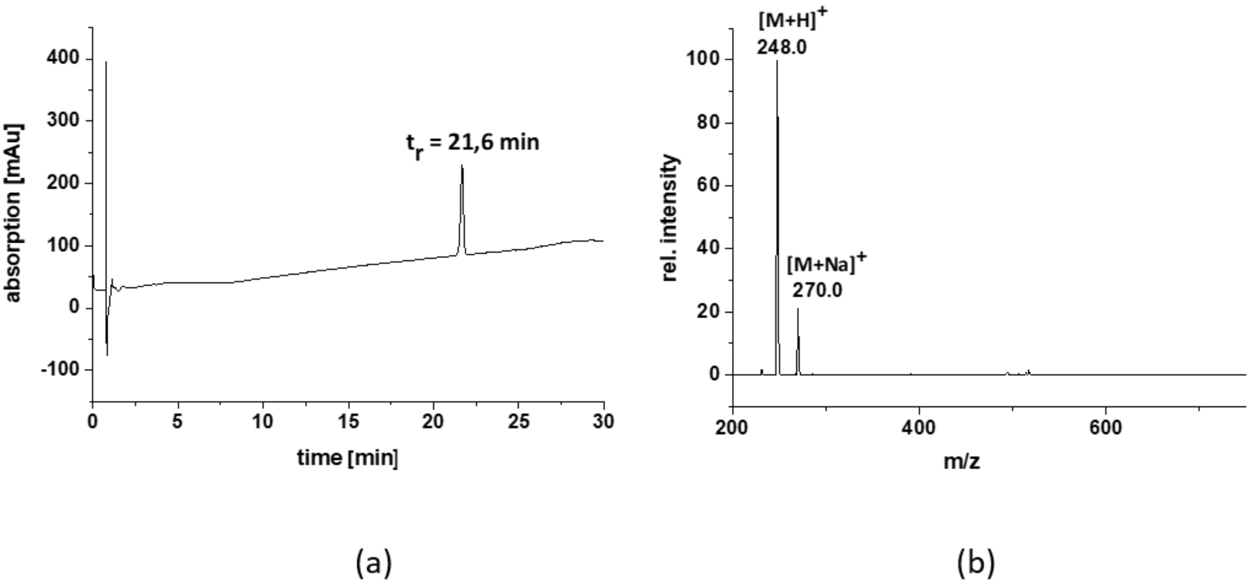

Figure S5. (a) LC-spectrum of CAA; (A: 95% H2O/ 5% MeCN/ 0.1% formic acid; 100% A -> 50% A in 30 min); (b) ESI-MS-spectrum of CAA.

ESI-MS for $C_{14}H_{17}NO_3$: $[M+H]^+$ calculated 248.29, measured 248.0.

N-(2-amino-2-oxoethyl)acrylamide

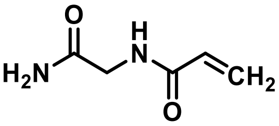

The synthesis procedure of the PA monomer *N*-(2-amino-2-oxoethyl)acrylamide is known from literature[2] and was carried out following it. 10 g glycinamide hydrochloride 2 (90.50 mmol) and 25 g potassium carbonate (181.00 mmol, 2.00 eq.) were dissolved in water [1.50 mL/mmol] and cooled to 0°C (ice bath). 8.8 mL of acryloyl chloride 5 (108.60 mmol, 1.20 eq.) in diethyl ether [2.50 mL/mmol] was added slowly (about 30 min). After addition was complete, the ice bath was removed and the reaction solution was stirred for 2 h at RT. The organic phase was removed on the rotary evaporator and the potassium carbonate was precipitated by addition of 1 L of cold acetone and filtered off. Acetone was removed on the rotary evaporator and a spatula tip of hydroquinone was added to the aqueous phase as a polymerization inhibitor. Now the aqueous phase could be removed on the rotary evaporator and the product was obtained as a colorless solid with a yield of 74 %.

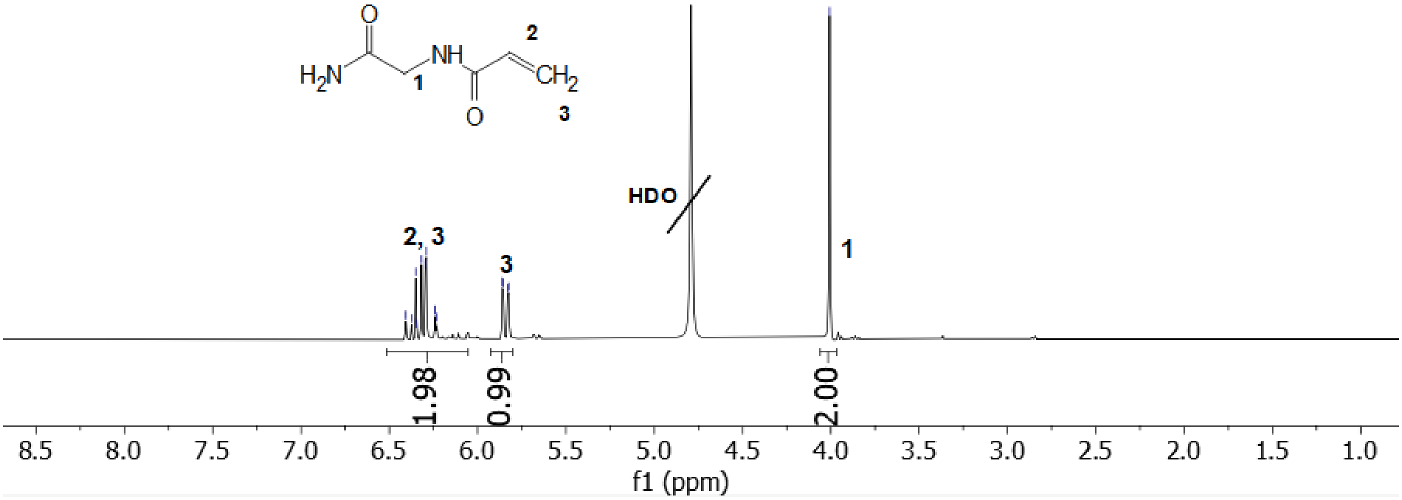

Figure S6. ^{1}H NMR spectrum (300 MHz, D_2O) of *N*-(2-amino-2-oxoethyl)acrylamide.

^{1}H NMR (300 MHz, D_2O) δ [ppm] 6.51-6.16 (m, 2H, -C**H**=C**H**$_2$), 5.84 (dd, *J* = 9.6, 2.0 Hz, 1H, =C**H**$_2$), 4.01 (s, 2H, -C**H**$_2$-)

2. Determination of reaction conditions for free radical polymerization

First, **HY** was homopolymerized to determine the basic reaction conditions for free radical polymerization of the Mfp-inspired copolymers (Scheme S1, Table S1). As expected, the average molecular weight increased by increasing the monomer concentration. Similarly, increasing the amount of initiator led to increased dispersity and lowering of the average molecular weight. Good results were obtained with an initiator amount of 1 mol% AIBN, so this was used for further copolymerizations. With 0.5 mol% initiator, no average molecular weight could be determined, since presumably the amount of initiator was too low for the reaction, or the inhibitor added to commercially purchased monomers stopped the polymerization early. With higher amounts of initiator, the measured molecular weights were too low.

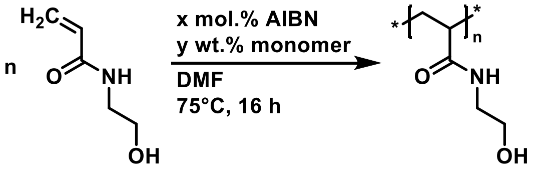

Scheme S1. Reaction scheme for the synthesis of the **HY**-homopolymer.

Table S1. Reaction conditions for homopolymerization of the HY monomer.

#	AIBN [mol%]	[Monomer] [wt.%]	$\bar{M}_n$[a] [kDa]	Đ[a]
1	0.5	5	-	-
2	1	5	6.8	1.35
3	2	5	-	-
4	3	5	4.9	1.50
5	1	10	4.1	1.40
6	1	20	14.0	1.90

[a]: determined by SEC

Subsequently, it was verified which monomer concentration leads to good results for a simplified copolymer system of **TA** and **HY** (Scheme S2, Table S2). At higher concentrations, the average molecular weight increased and at lower concentrations it decreased as expected. Since the actual incorporation of the monomers deviated only slightly from the theoretical values, and inaccuracies are to be expected in a determination by ^1H NMR-spectroscopy, a 1:1 incorporation of the monomers can be assumed. For the following copolymerizations, 20 wt.% monomer and 1 mol% initiator were set as reaction conditions.

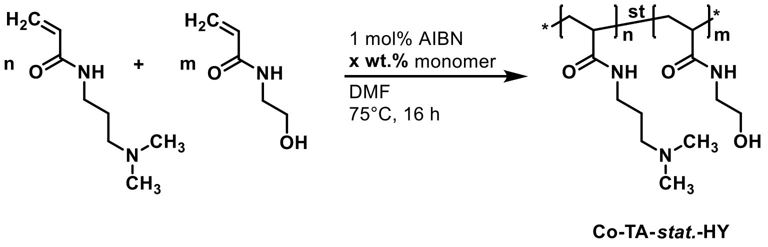

Scheme S2. Reaction scheme for the synthesis of the **TA-HY**-copolymers.

Table S2. Reaction conditions for copolymerization of **TA** and **HY**.

#	monomer ratio HY eq.	TA eq.	monomer concentration [wt.%]	theor. TA incorporation [%]	determ. TA incorporation [%][b]	Mn[a] [kDa]	Đ[a]
1	1	1	5	50	58	-	-
2	3	1	5	25	28	5.6	1.30
3	6	1	5	14	15	9.0	1.60
4	1	1	20	50	53	-	-
5	3	1	20	25	28	34.0	1.23
6	6	1	20	14	17	40.0	1.80
7	6	1	10	14	-	9.4	1.45
8	6	1	30	14	-	37.0	1.65
9	6	1	50	14	-	-	-

[a]: determined by SEC, [b]: determined by ^{1}H NMR-spectroscopy

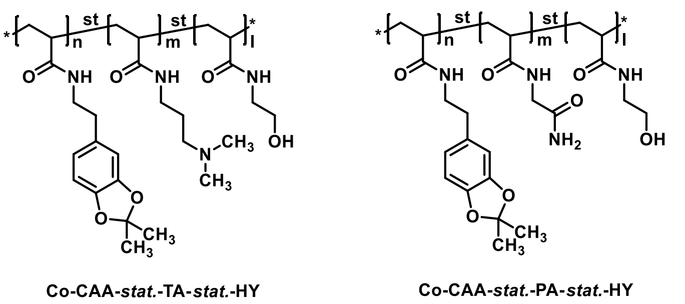

Scheme S1 Copolymers synthesized to determine optimal polymerization conditions.

The polymer composition was analyzed during the polymerization reaction to estimate the distribution of the different monomers in the final polymers. Two polymers CAA-*stat.*-TA-*stat.*-HY and CAA-*stat.*-PA-*stat.*-HY were polymerized and analyzed at different intervals during the reaction. The reactions were carried out with a theoretical monomer incorporation of 5% CAA, 45% TA and 50% HY (CAA-*stat.*-TA-*stat.*-HY) and 5% CAA, 45% PA and 50% HY (CAA-*stat.*-PA-*stat.*-HY). Monomers were placed in a microwave tube and dissolved in DMF. The reaction solution was degassed for 20 min with inert gas (N_2) and stirred for 18 h at 75°C. At the different intervals of 30 min, 1 h, 2 h, 4 h, and overnight, samples were taken from the reaction solution and the reaction was stopped by precipitation in diethyl ether. Subsequently, the obtained copolymers were dialyzed (2 kDa exclusion size) and lyophilized. ^{1}H NMR-spectroscopy was used to determine the monomer composition (Table S3).

Table S3. Obtained monomer incorporation in the copolymer CAA-TA-HY.

	monomer incorporation [%][a]					
	**CAA-*stat.*-TA-*stat.*-HY**			**CAA-*stat.*-PA-*stat.*-HY**		
	**CAA**	**TA**	**HY**	**CAA**	**PA**	**HY**
30 min	6	51	43	4.5	43	52.5
60 min	4	53	43	5.5	38	56.5
120 min	3	56	41	6.5	39	54.5
240 min	3	54	43	-	-	-
over night	2.5	53.5	44	-	-	-

[a]: determined by ^1H NMR-spectroscopy

3. Synthesis and characterization of final polymers

PA50-*stat.*-HY50

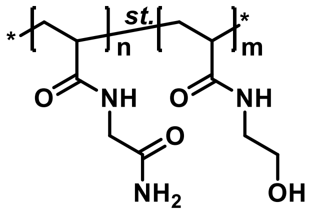

1.54 g of *N*-acryloylglycinamide (12.00 mmol), 1.38 g of *N*-hydroxyethylacrylamide (12.00 mmol, 1.00 eq.), and 39.41 mg of AIBN (1 mol%) were placed in a microwave tube and dissolved in DMF. The reaction solution was degassed for 20 min with inert gas (N_2) and stirred for 18 h at 75°C. The mixture was then poured onto diethyl ether to precipitate the polymer. After centrifugation for 5 min and decantation of the ether, the polymer was dried under a nitrogen atmosphere. It was then dissolved in water and dialyzed with a dialysis tube with a MWCO of 7.50 kDa for five cycles. After subsequent lyophilization, the polymer PA50-*stat.*-HY50 was obtained as a colorless solid with a yield of 39%.

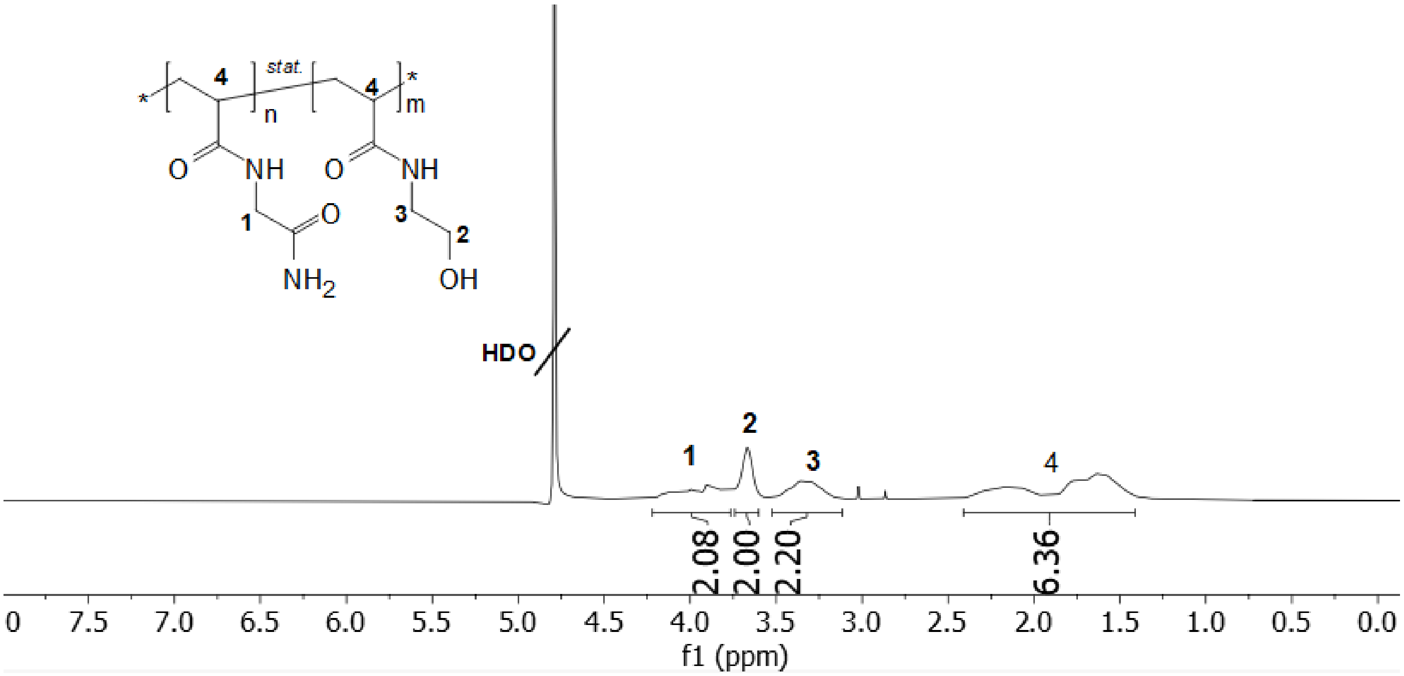

Figure S7. ^1H NMR spectrum (300 MHz, D_2O) of **PA50-*stat.*-HY50**.

^1H NMR (300 MHz, D_2O) δ [ppm] 3.94 (m, **1**), 3.67 (s, **2**), 3.34 (s, **3**), 2.47-1.36 (m, **4**).

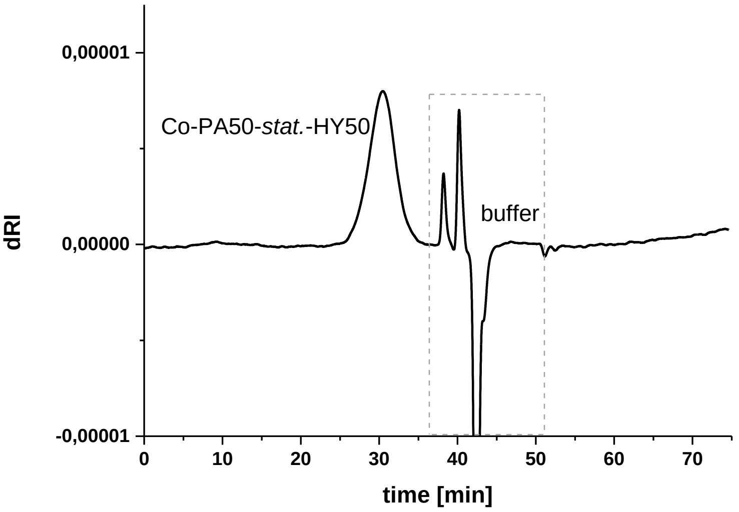

Figure S8. H_2O SEC-MALS spectrum of **PA50-*stat.*-HY50**.

SEC-MALS: $\overline{M}_n$ = 50.33 kDa; Đ = 1.36

TA52-*stat.*-HY48

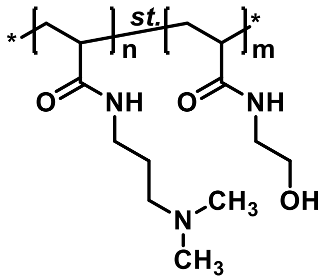

1.64 mL of dimethylaminopropylacrylamide (10.00 mmol), 1.151 g of *N*-hydroxyethylacrylamide (10.00 mmol, 1.00 eq.), and 32.84 mg of AIBN (1 mol%) were placed in a microwave tube and dissolved in 14.3 mL of DMF. The reaction solution was degassed for 20 min with inert gas (N_2) and stirred for 18 h at 75°C. The solution was then poured onto diethyl ether, which was acidified with 0.5 mL trifluoroacetic acid, to precipitate the polymer. After centrifugation for 5 min and decantation of the ether, the polymer was dried under a nitrogen atmosphere. It was then dissolved in a little water and dialyzed with a dialysis tube with a MWCO of 7.50 kDa for five cycles. After subsequent lyophilization, the polymer TA52-*stat.*-HY48 was obtained as a colorless solid with a yield of 91%.

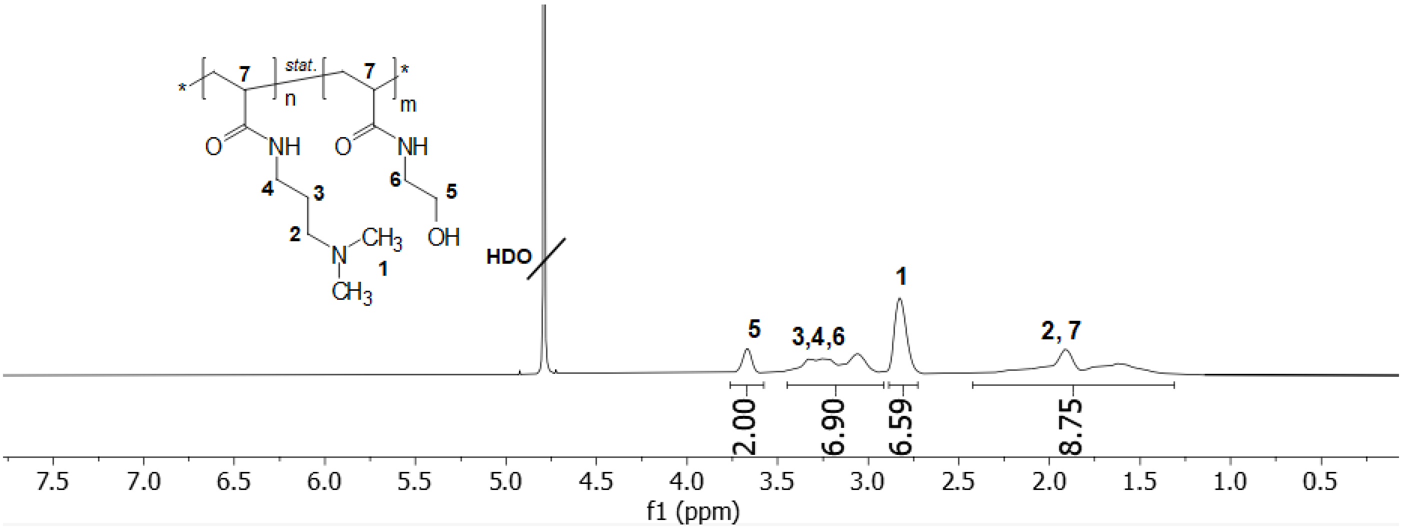

Figure S9. ^{1}H NMR spectrum (300 MHz, D_2O) of **TA52-*stat.*-HY48**.

^{1}H NMR (300 MHz, D_2O) δ [ppm] 3.67 (s, **5**), 3.53-2.94 (m, **3**, **4**, **6**), 2.82 (s, **1**), 2.41-1.28 (m, **7**, **2**).

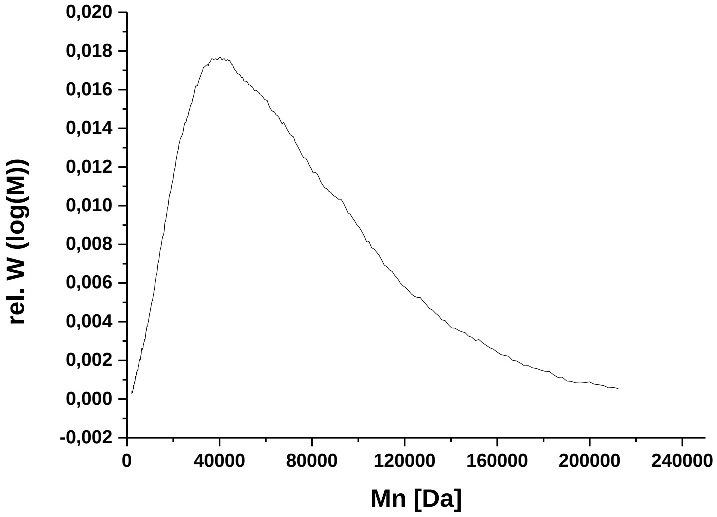

Figure S10. DMAC-SEC spectrum of **TA52-*stat.*-HY48**.

SEC: $\overline{M}_n$ = 23.5 kDa; Đ = 1.97

CAA4-*stat.*-TA48-*stat.*-HY48

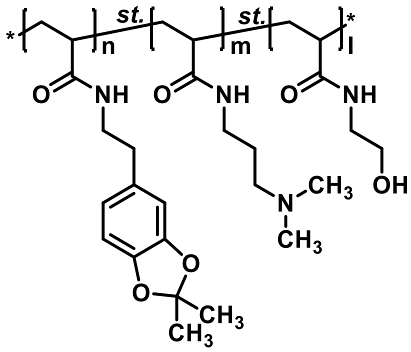

0.247 g CAA monomer (1.00 mmol), 1.48 mL dimethylaminopropylacrylamide (9.00 mmol, 9.00 eq.), 1.15 g *N*-hydroxyethylacrylamide (10.00 mmol, 10.00 eq.), and 32.84 mg AIBN (1 mol%) were placed in a microwave tube and dissolved in DMF. The reaction solution was degassed for 20 min with inert gas (N_2) and stirred for 18 h at 75°C. Then, the solution was poured onto diethyl ether, which was acidified with 0.5 mL of trifluoroacetic acid, thus precipitating the polymer. After centrifugation for 5 min and decantation of the ether, the polymer was dried under a nitrogen atmosphere. It was then dissolved in water and dialyzed with a dialysis tube with a MWCO of 7.50 kDa for five cycles. After subsequent lyophilization, the polymer CAA4-*stat.*-TA48-*stat.*-HY48 was obtained as a yellowish solid with a yield of 98%. The monomer incorporation was verified by ^{1}H NMR spectroscopy.

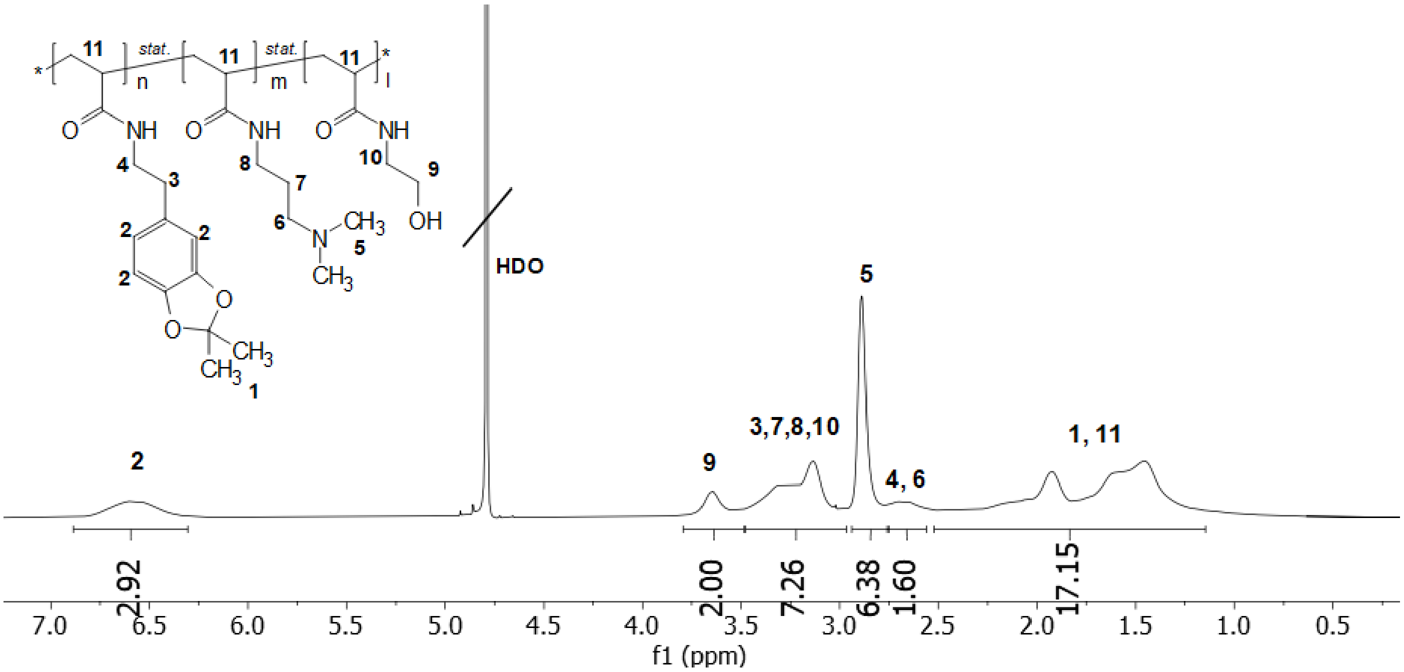

Figure S1. ^{1}H NMR spectrum (300 MHz, D_2O) of **CAA4-*stat.*-TA48-*stat.*-HY48**.

^{1}H NMR (300 MHz, D_2O) δ [ppm] 6.79 (s, **2**), 3.67 (s, **9**), 3.52-3.05 (m, **3**, **7** ,**8**, **10**), 2.89 (s, **5**), 2.76-2.54 (s, **6**, **4**), 2.45-1.29(m, **1**, **11**).

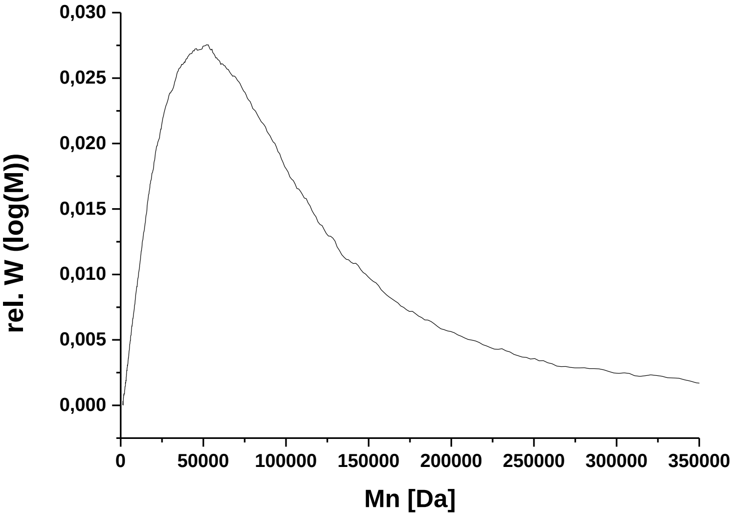

Figure S2. DMAC-SEC spectrum of **CAA4-*stat.*-TA48-*stat.*-HY48.**

SEC: $\overline{M}_n$ = 21.9 kDa; Đ = 2.61

CAA3-*stat.*-PA45-*stat.*-HY52

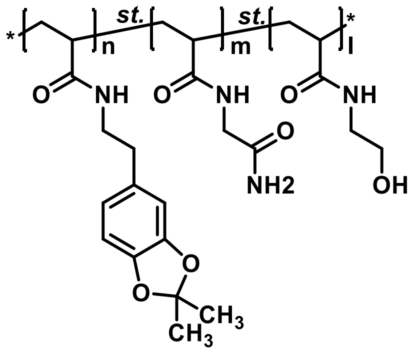

0.247 g CAA monomer (1.00 mmol), 1.15 g *N*-acryloylglycinamide (9.00 mmol, 9.00 eq.), 1.15 g *N*-hydroxyethylacrylamide (10.00 mmol, 10.00 eq.), and 32.84 mg AIBN (1 mol%) were placed in a microwave tube and dissolved in DMF (20 wt.%). The reaction solution was degassed for 20 min with inert gas (N_2) and stirred for 18 h at 75°C. Then, the solution was poured onto diethyl ether, thus precipitating the polymer. After centrifugation for 5 min and decantation of the ether, the polymer was dried under a nitrogen atmosphere. It was then dissolved in water and dialyzed with a dialysis tube with a MWCO of 7.50 kDa for five cycles. After subsequent lyophilization, the polymer CAA4-*stat.*-PA45-*stat.*-HY52 was obtained as a yellowish solid with a yield of 45%. The monomer incorporation was verified by ^{1}H NMR spectroscopy.

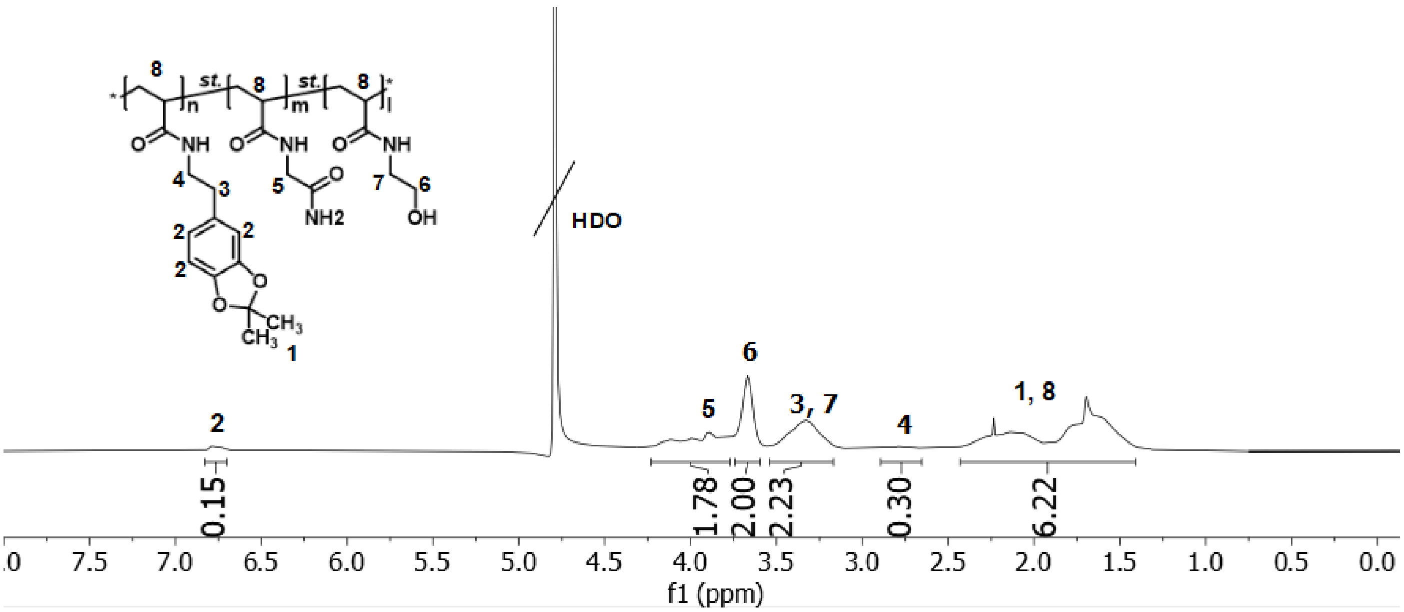

Figure S3. ^{1}H NMR spectrum (300 MHz, D_2O) of **CAA3-*stat.*-PA42-*stat.*-HY52**.

^{1}H NMR (300 MHz, D_2O) δ [ppm] δ [ppm] 6.77 (s, **2**), 4.32-3.76 (m, **5**), 3.67 (s, **6**), 3.51-3.15 (m, **3**, **7**), 2.75 (s, **4**), 2.60-1.36(m, **1, 8**).

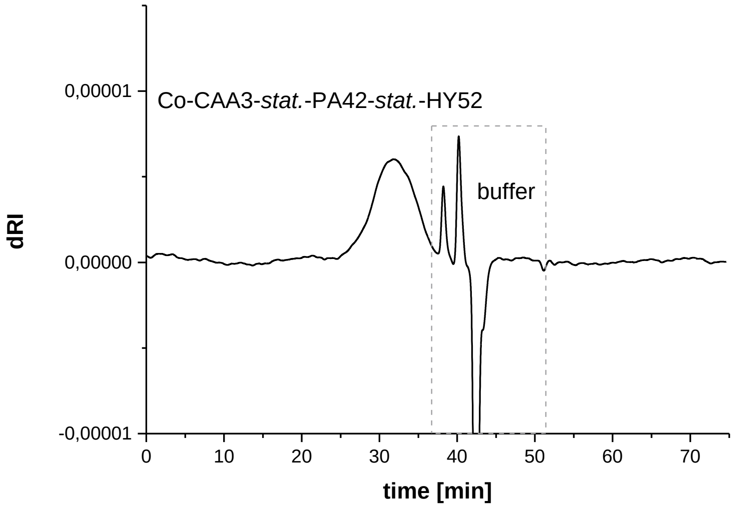

Figure S4. H_2O-SEC-MALS spectrum of **CAA3-*stat.*-PA42-*stat.*-HY52.**

SEC-MALS: $\overline{M}_n$ = 23.4 kDa; Đ = 1.92

TA15-*stat.*-PA13-*stat.*-HY72

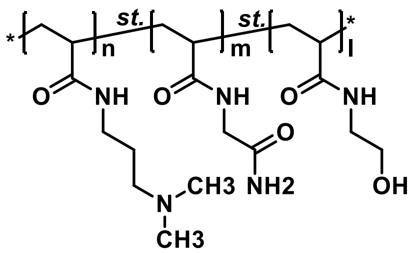

0.493 mL dimethylaminopropylacrylamide (3.00 mmol), 0.384 g *N*-acryloylglycinamide (3.00 mmol, 1.00 eq.), 1.612 g *N*-hydroxyethylacrylamide (14.00 mmol, 4.70 eq.), and 32.84 mg AIBN (1 mol%) were placed in a microwave tube and dissolved in DMF (20 wt.%). The reaction solution was degassed for 20 min with inert gas (N_2) and stirred for 18 h at 75°C. Then, the solution was poured onto diethyl ether, which was acidified with 0.5 mL of trifluoroacetic acid, thus precipitating the polymer. After centrifugation for 5 min and decantation of the ether, the polymer was dried under a nitrogen atmosphere. It was then dissolved in water and dialyzed with a dialysis tube with a MWCO of 7.50 kDa for five cycles. After subsequent lyophilization, the polymer TA15-*stat.*-PA13-*stat.*-HY72 was obtained as a colorless solid with a yield of 93%. The monomer incorporation was verified by ^1H NMR spectroscopy.

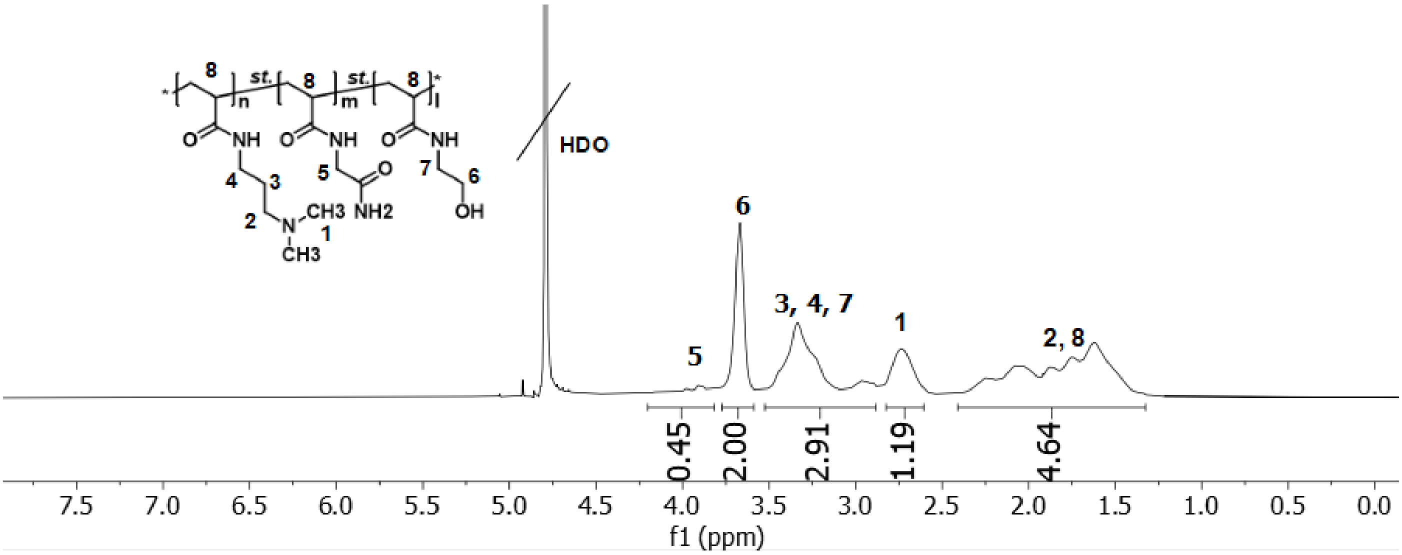

Figure S5. ^1H NMR spectrum (300 MHz, D_2O) of **TA15-*stat.*-PA13-*stat.*-HY72**.

^1H NMR (300 MHz, D_2O) δ [ppm] δ [ppm] 4.32-3.84(m, **5**), 3.67 (s, **6**), 3.33 (m, **3**, **4**, **7**), 2.73 (s, **1**), 2.41-1.37 (m, **2**, **8**).

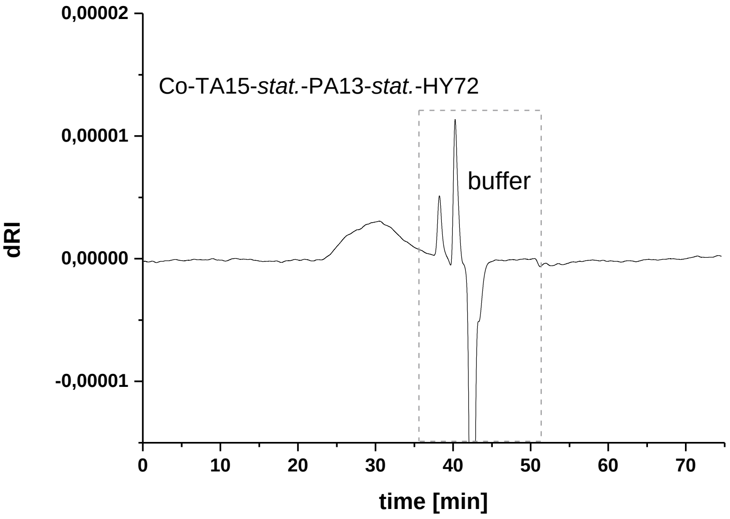

Figure S6. H_2O-SEC-MALS spectrum of **TA15-*stat.*-PA13-*stat.*-HY72.**

SEC-MALS: $\overline{M}_n$ = 69.93 kDa; Đ = 1.74

CAA-*stat.*-TA-*stat.*-PA-*stat.*-HY

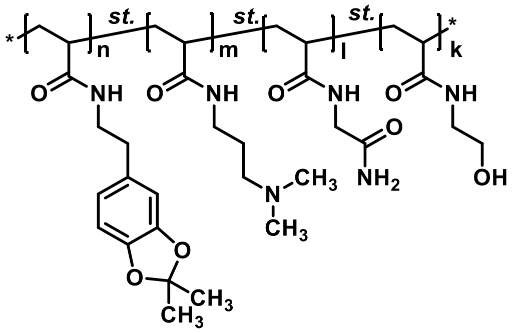

CAA monomer (1.0 eq.), dimethylaminopropylacrylamide (TA monomer, **x** eq., see Table S4), *N*-acryloylglycinamide (PA monomer, **y** eq., see Table S4), *N*-hydroxyethylacrylamide (HY monomer, **z** eq., see Table S4), and AIBN (1 mol%) were placed in a microwave tube and dissolved in DMF (20 wt.%). The reaction solution was degassed for 20 min with inert gas (N_2) and stirred for 18 h at 75°C. Then, the solution was poured onto diethyl ether, which was acidified with 0.5 mL of trifluoroacetic acid, thus precipitating the polymer. After centrifugation for 5 min and decantation of the ether, the polymer was dried under a nitrogen atmosphere. It was then dissolved in water and dialyzed with a dialysis tube with a MWCO of 7.50 kDa for five cycles. After subsequent lyophilization, the polymer TA15-*stat.*-PA13-*stat.*-HY72 was obtained as a colorless solid with a yield of 93%. The monomer incorporation was verified by ^{1}H NMR spectroscopy.

Table S4. Used quantities and equivalents build the final polymers by incorporation of all four monomers.

polymer	Used equivalents CAA	TA	PA	HY	yield [%]
**CAA4- *stat.*-TA22-*stat.*-PA17-*stat.*-HY57**	1.00 (0.247 g)	4.50 (0.740 mL)	4.50 (0.576 g)	10.00 (1.151 g)	85
**CAA13-*stat.*-TA15-*stat.*-PA18-*stat.*-HY54**	1.00 (0.742 g)	1.00 (0.493 mL)	1.00 (0.384 g)	3.70 (1.266 g)	91
**CAA5-*stat.*-TA5-*stat.*-PA7-*stat.*-HY83**	1.00 (0.247 g)	1.00 (0.164 mL)	1.00 (0.128 g)	17.00 (1.957 g)	94

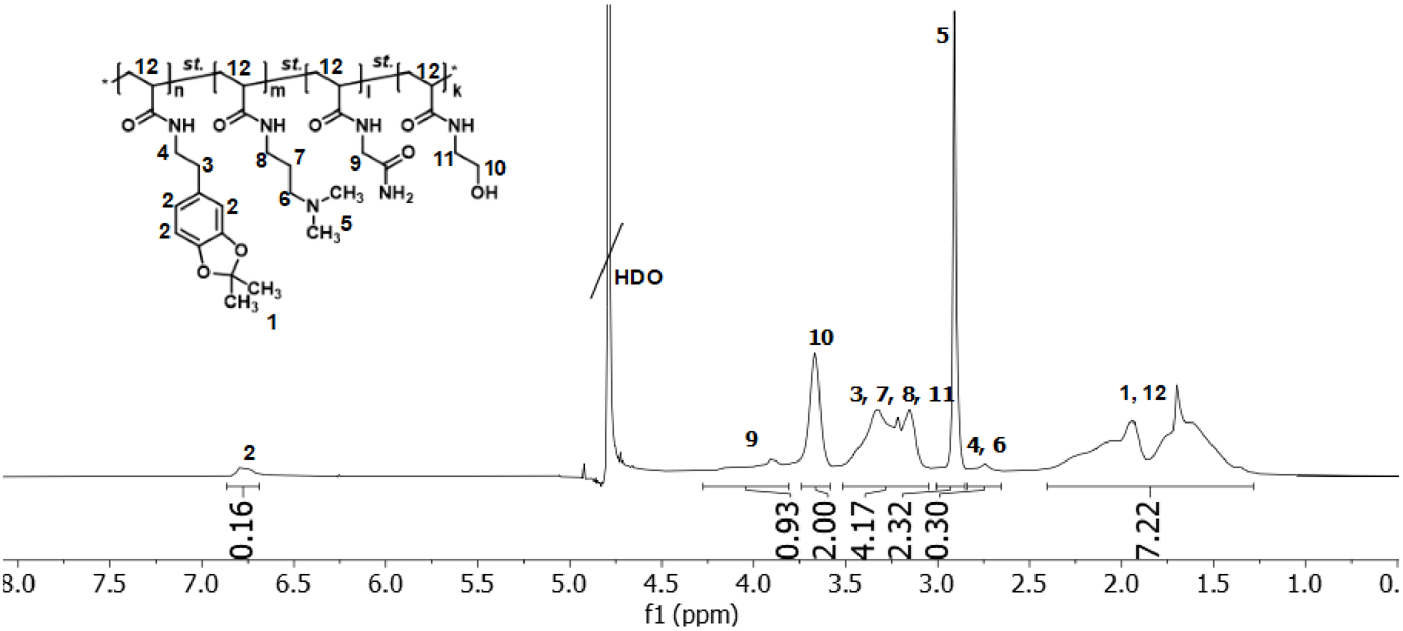

Figure S7. ^{1}H NMR spectrum (300 MHz, D_2O) of **CAA4-*stat.*-TA22-*stat.*-PA17-*stat.*-HY57**.

[1]**H NMR (300 MHz, D_2O)** δ [ppm] δ [ppm] 6.77 (s, **2**), 4.35-3.80 (m, **9**), 3.67 (s, **10**), 3.48-3.06(m, **3**, **7**, **8**, **11**), 2.91 (s, **5**), 2.70 (s, **4**, **6**), 2.51-1.24 (m, **1**, **12**).

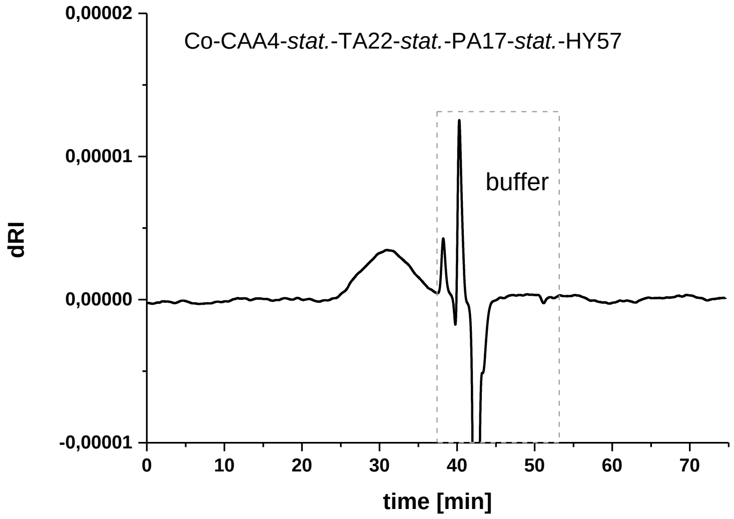

Figure S8. H_2O-SEC-MALS spectrum of **CAA4-*stat.*-TA22-*stat.*-PA17-*stat.*-HY57.**

SEC-MALS: $\overline{M}_n$ = 64.43 kDa; Đ = 1.73

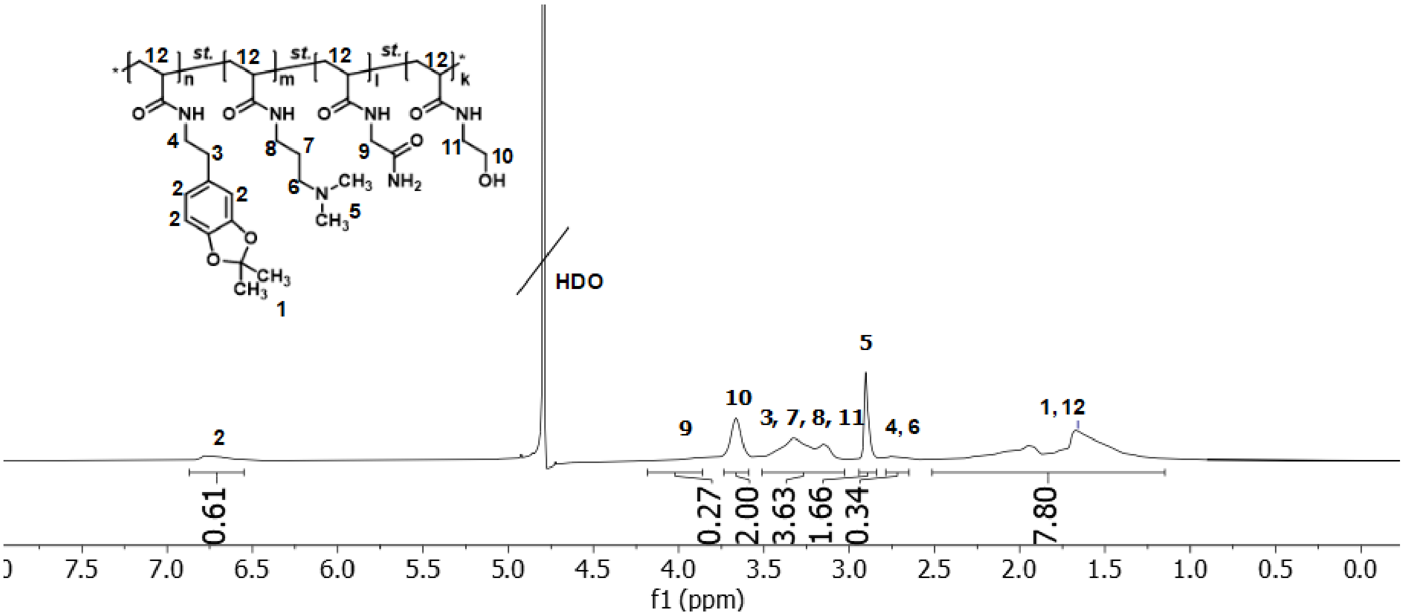

Figure S9. ^{1}H NMR spectrum (300 MHz, D_2O) of **CAA13-*stat.*-TA15-*stat.*-PA18-*stat.*-HY54**.

^{1}H NMR (300 MHz, D_2O) δ [ppm] δ [ppm] 6.75 (s, **2**), 4.20-3.82 (m, **9**), 3.66 (s, **10**), 3.52-3.04 (m, **3**, **7**, **8**, **11**), 2.90 (s, **5**), 2.73 (s, **4**, **6**), 2.48-1.31 (m, **1**, **12**).

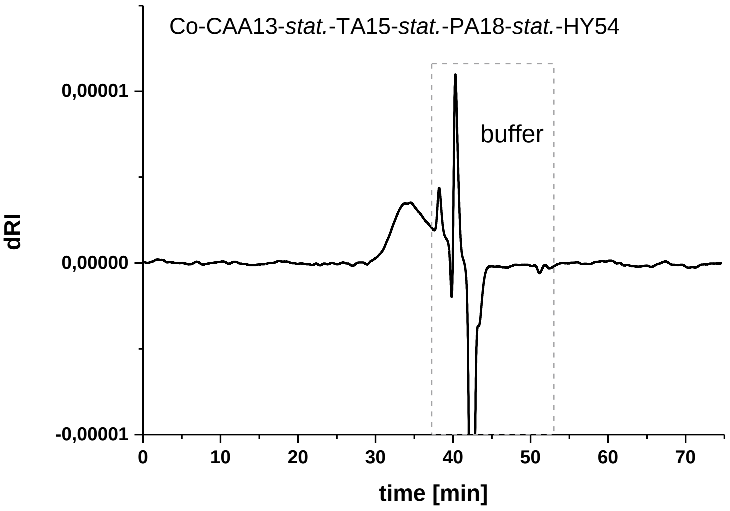

Figure S20. H_2O-SEC-MALS spectrum of **CAA13-*stat.*-TA15-*stat.*-PA18-*stat.*-HY54.**

SEC-MALS: $\overline{M}_n$ = 50.90 kDa; Đ = 1.12

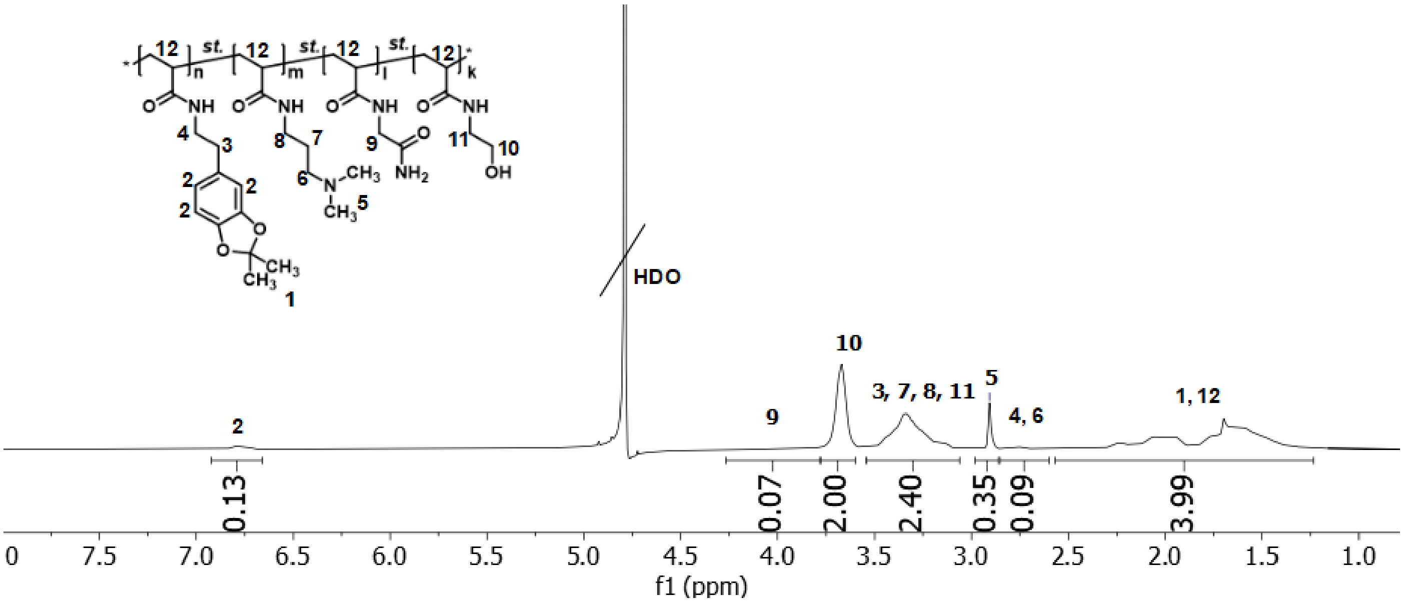

Figure S10. ^{1}H NMR spectrum (300 MHz, D_2O) of **CAA5-*stat.*-TA5-*stat.*-PA7-*stat.*-HY83**.

^{1}H NMR (300 MHz, D_2O) δ [ppm] δ [ppm] 6.77 (s, **2**), 4.23-3.83 (m, **9**), 3.67 (s, **10**), 3.52-3.06 (s, **3**, **7**, **8**, **11**), 2.91 (s, **5**), 2.74 (s, **4**, **6**), 2.47-1.26 (m, **1**, **12**).

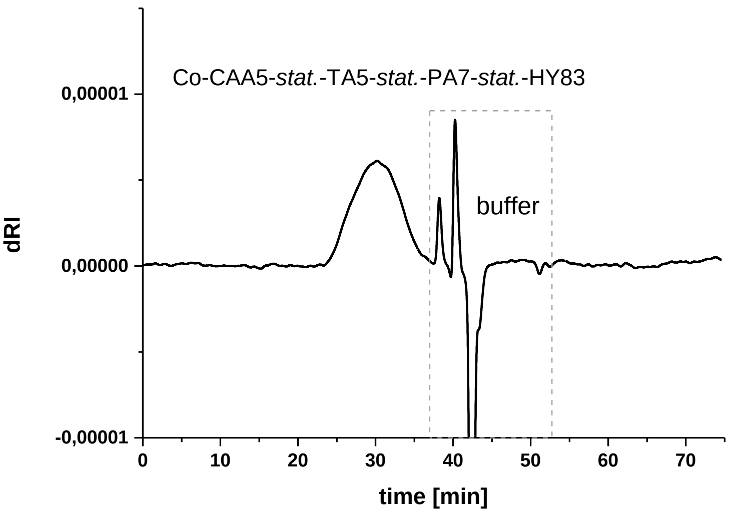

Figure S11. H_2O-SEC-MALS spectrum of **CAA5-*stat.*-TA5-*stat.*-PA7-*stat.*-HY83.**

SEC-MALS: $\overline{M}_n$ = 63.10 kDa; Đ = 1.87

4. Acetonide deprotection of final polymers

In order to obtain catechol units for adhesion studies, the acetonide protecting group was removed from the CAA monomers. The reaction was carried out in a mixture of TFA/H_2O (95:5) and the solution was stirred for 3 h at room temperature. After work-up by precipitation in diethyl ether followed by lyophilization, the polymers were stored under an inert gas atmosphere.

CAA4-*stat.*-TA48-*stat.*-HY48

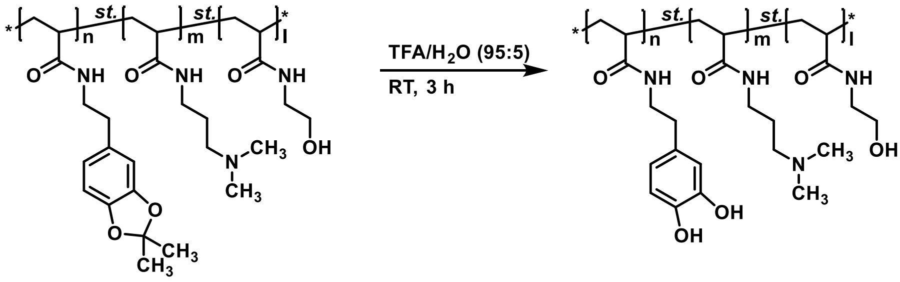

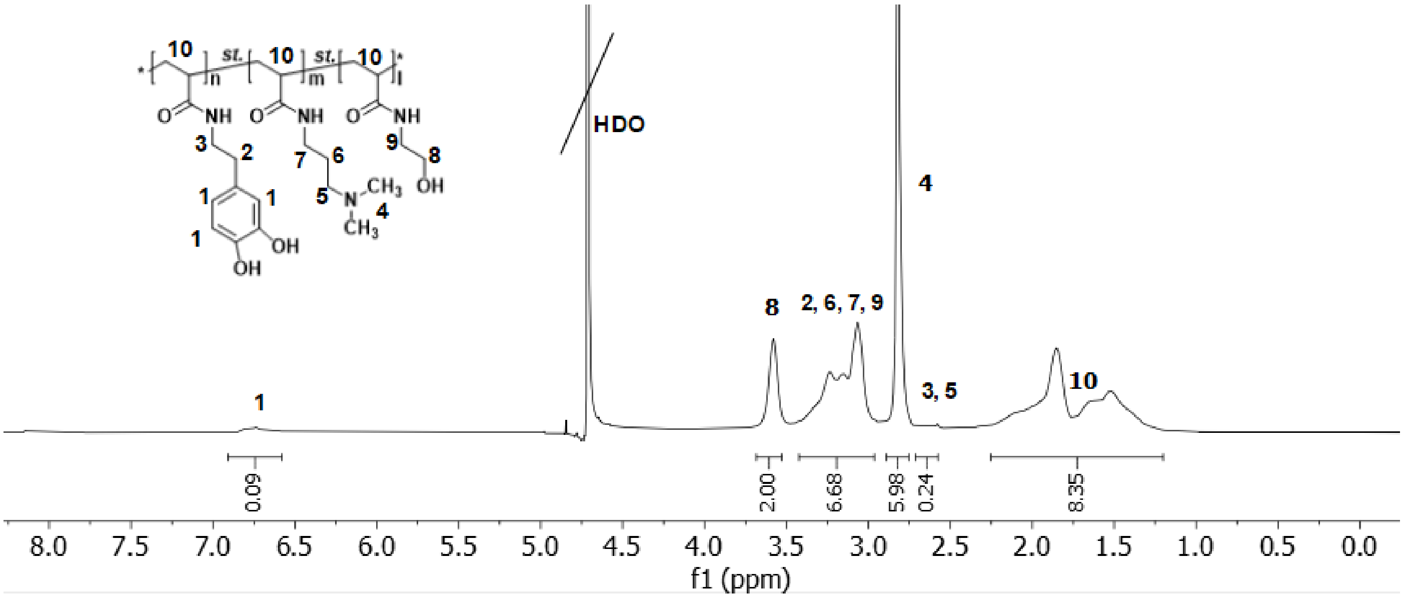

Figure S12. ¹H NMR spectrum (300 MHz, D_2O) of **Co-CA4-*stat.*-TA48-*stat.*-HY48**.

CAA3-*stat.*-PA45-*stat.*-HY52

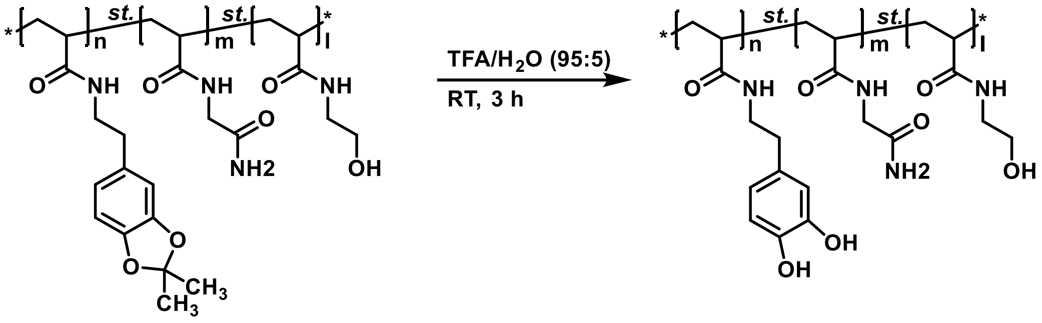

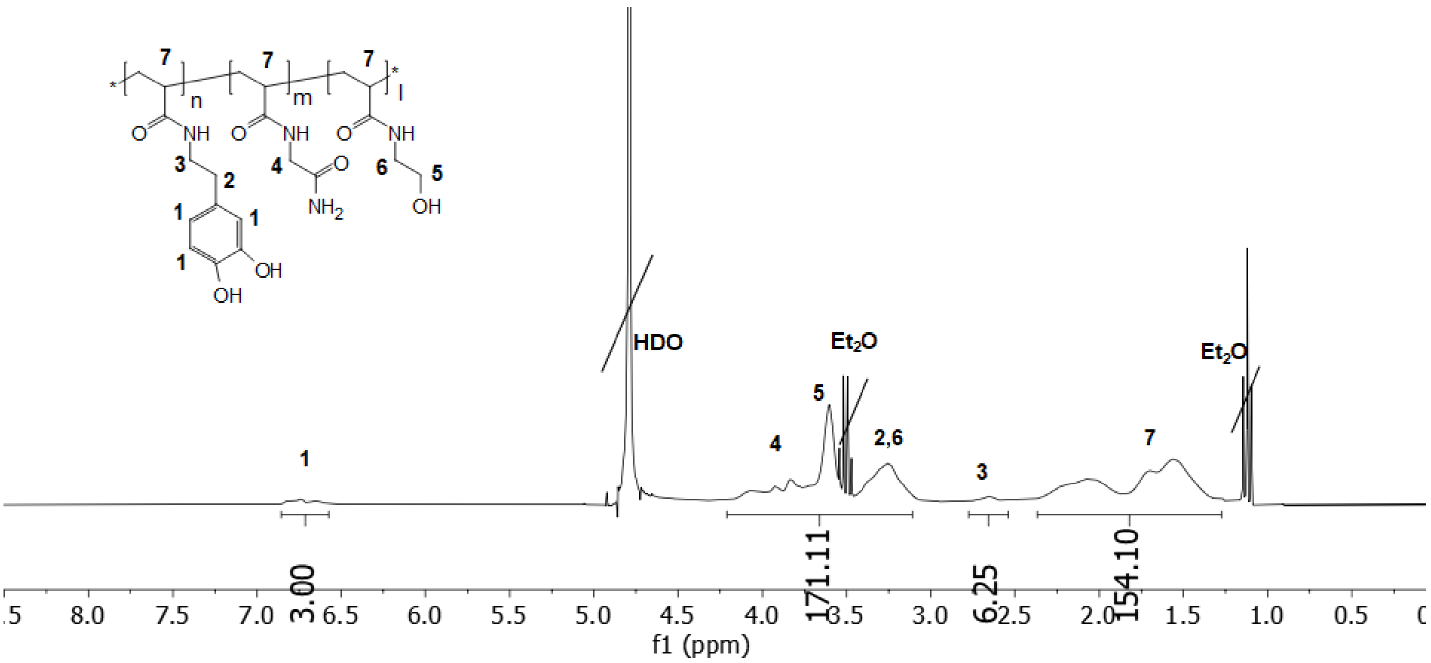

Figure S13. ^{1}H NMR spectrum (300 MHz, D_2O) of **Co-CA3-*stat.*-TA45-*stat.*-HY52**.

CAA4-*stat.*-TA22-*stat.*-PA17-*stat.*-HY57

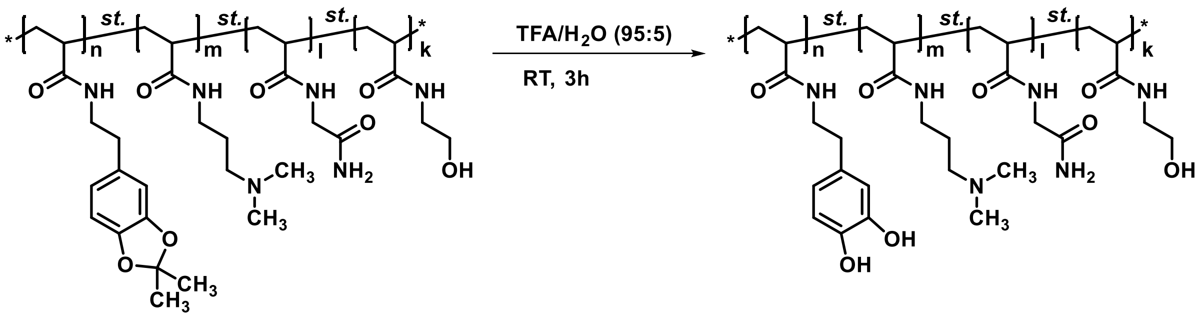

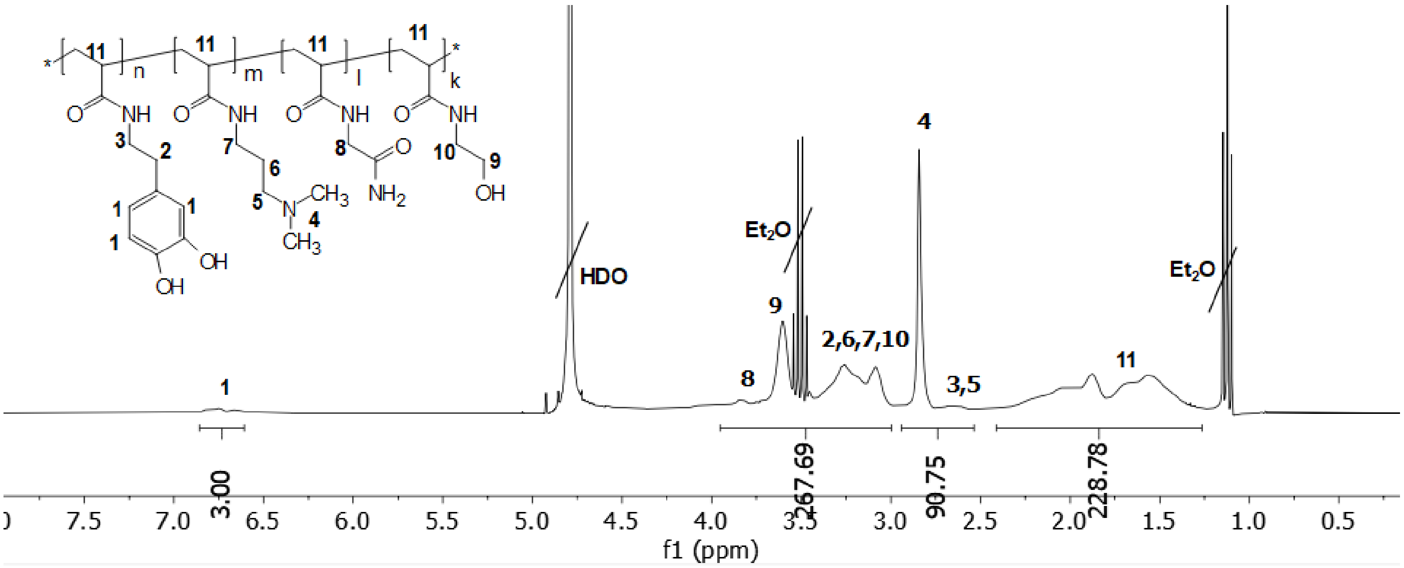

Figure S14. ^{1}H NMR spectrum (300 MHz, D_2O) of **CAA4-*stat.*-TA22-*stat.*-PA17-*stat.*-HY57.**

CAA13-*stat.*-TA15-*stat.*-PA18-*stat.*-HY54

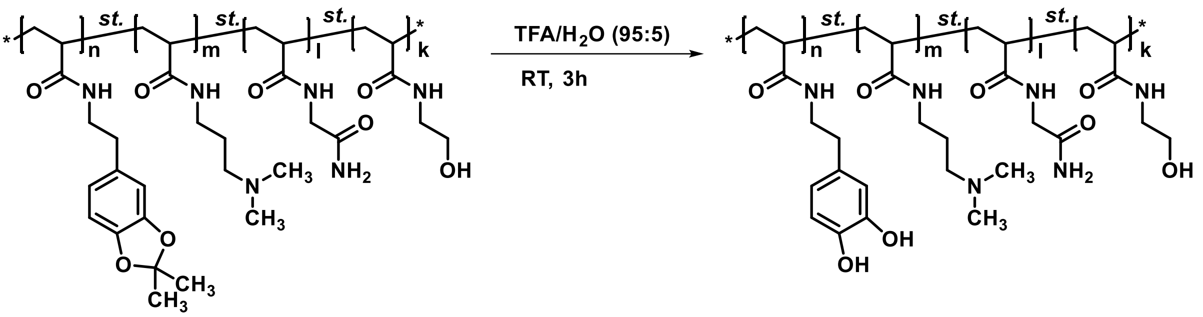

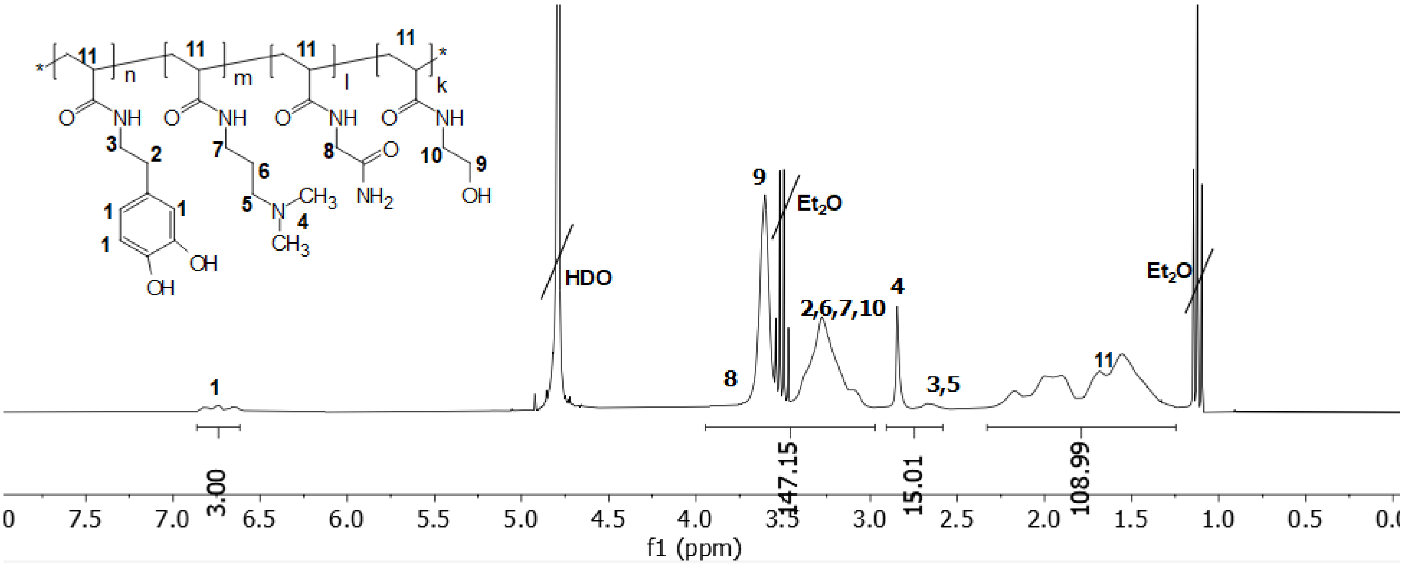

Figure S15. ^{1}H NMR spectrum (300 MHz, D_2O) of **CAA13-*stat.*-TA15-*stat.*-PA18-*stat.*-HY54.**

CAA5-*stat.*-TA5-*stat.*-PA7-*stat.*-HY83

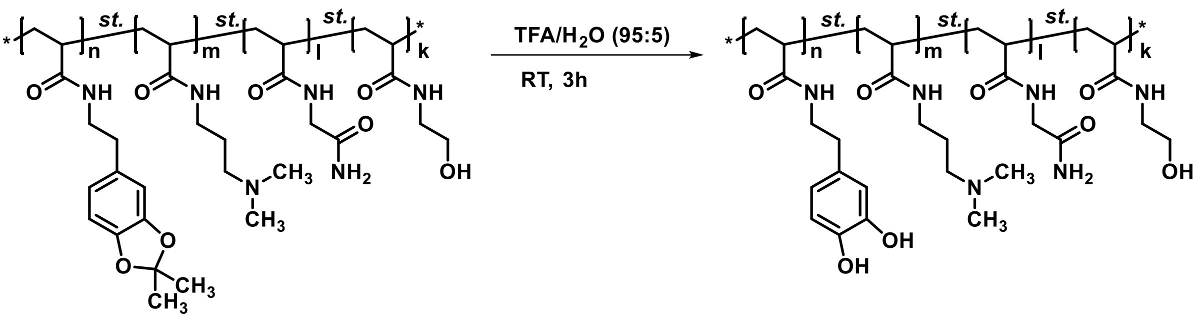

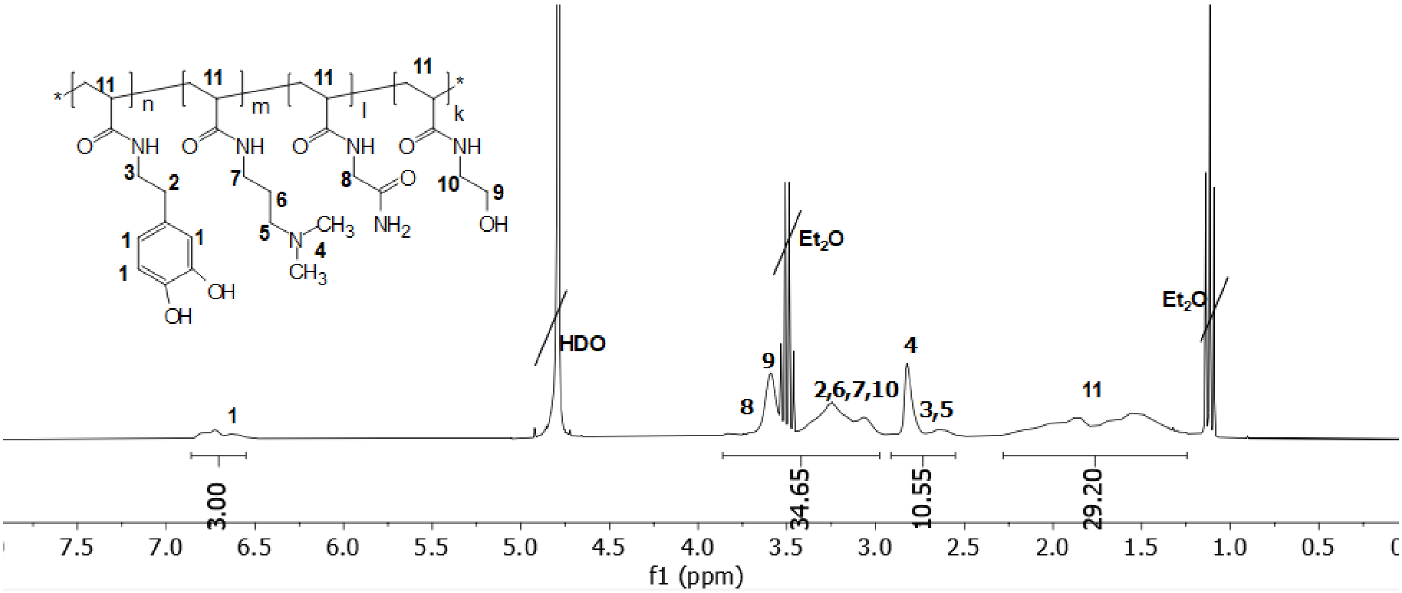

Figure S16. ^{1}H NMR spectrum (300 MHz, D_2O) of **CAA5-*stat.*-TA5-*stat.*-PA7-*stat.*-HY83.**

References

1. Liu, Z.; Hu, B.-H.; Messersmith, P.B. Acetonide protection of dopamine for the synthesis of highly pure N-docosahexaenoyldopamine. *Tetrahedron Lett.* **2010**, *51*, 2403-2405, doi:https://doi.org/10.1016/j.tetlet.2010.02.089.
2. Glatzel, S.; Badi, N.; Päch, M.; Laschewsky, A.; Lutz, J.-F. Well-defined synthetic polymers with a protein-like gelation behavior in water. *Chem. Commun.* **2010**, *46*, 4517-4519, doi:10.1039/C0CC00038H.
